# Revealing the Impact of Oxygen Dissolved in Electrolytes on Aqueous Zinc-Ion Batteries

**DOI:** 10.1016/j.isci.2020.100995

**Published:** 2020-03-20

**Authors:** Lijun Su, Lingyang Liu, Bao Liu, Jianing Meng, Xingbin Yan

**Affiliations:** 1 Laboratory of Clean Energy Chemistry and Materials, Lanzhou Institute of Chemical Physics, Chinese Academy of Sciences, Lanzhou 730000, China; 2 Center of Materials Science and Optoelectronics Engineering, University of Chinese Academy of Sciences, Beijing 100049, China; 3 Cuiying Honors College, Lanzhou University, Lanzhou, 730000, China; 4 Dalian National Laboratory for Clean Energy, Chinese Academy of Sciences, Dalian 116023, China

**Keywords:** Electrochemical Energy Storage, Energy Storage, Materials Characterization

## Abstract

Aqueous zinc-ion batteries (ZIBs) are promising low-cost and high-safety energy storage devices. However, their capacity decay especially at the initial cyclic stage is a serious issue. Herein, we reveal that the dissolved oxygen in aqueous electrolyte has significant impact on the electrochemistry of Zn anode and ZIBs. After removing oxygen, the symmetrical set-up of Zn/Zn is capable of reversible plating/stripping with a 20-fold lifetime enhancement compared with that in oxygen enrichment condition. Taking aqueous Zn-MnO_2_ battery as an example, although the presence of oxygen can contribute an extra capacity over 20% at the initial cycles due to the electrocatalytic activity of MnO_2_ with oxygen, the corrosion of Zn anode can be eliminated in the oxygen-free circumstance and thus offering a better reversible energy storage system. The impact of the dissolved oxygen on the cycling stability also exists in other ZIBs using vanadium-based compounds, Birnessite and Prussian blue analog cathodes.

## Highlights

• Dissolved oxygen in electrolyte has significant impact on Zn anode and ZIBs
• The dissolved oxygen caused the thermodynamically instability of Zn
• Oxygen dissolved in electrolyte accelerates the corrosion on Zn
• The ZIBs present better cycling stability after removing oxygen

## Graphical Abstract

**Figure:**
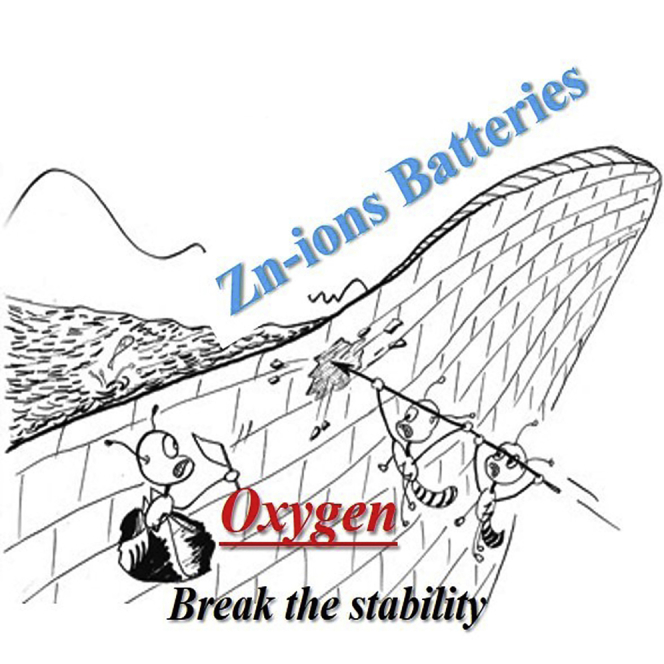

## Introduction

Aqueous zinc-ion batteries (ZIBs) have gained more and more attention mainly due to their low cost as well as high safety (Parker et al., 2017, Zheng et al., 2019), and significant research progress has been achieved in designing aqueous ZIBs with Zn anode and various cathode materials (Trócoli and La Mantia, 2015, Sun et al., 2017, Pan et al., 2018, Wan et al., 2019). However, the reported ZIBs generally suffer poor cycling stability especially at the initial cycles. Taking the reported ZIBs using the most typical ZnSO_4_ aqueous electrolytes as examples, whether the cathode material is vanadium oxides (Wan et al., 2018, Yang et al., 2018), manganese oxides (Mondoloni et al., 1992, Xu et al., 2012, Alfaruqi et al., 2015a, Alfaruqi et al., 2015b), Prussian blue analog materials (Zhang et al., 2015), and so on, all suffer significant capacity decay in the initial cycling stage (Table S1) (Fang et al., 2018, Song et al., 2018). Such decay was mainly explained because of the dissolution of cathode materials into the bulk electrolytes as well as the formation of Zn dendrite (Lee et al., 2014, Lee et al., 2016, Boeun et al., 2015, Mainar et al., 2018, Yi et al., 2018, Zhao et al., 2019b, Zhao et al., 2019a).

On the one hand, it is well known that metal Zn is easily oxidized in the air (Mainar et al., 2018, Yi et al., 2018, Zhao et al., 2019b, Zhao et al., 2019a). On the other hand, the dissolved oxygen is commonly more than 9 mg L^−1^ in low-concentration aqueous electrolytes under ambient temperature and pressure, as high as the oxygen content in the air (Davis et al., 1967, Benson et al., 1979, Luo et al., 2010). Therefore, the oxygen dissolved in the aqueous electrolytes of ZIBs might oxidize the Zn anode, thereby impacting the performance of the devices. However, the study on the influence of the dissolved oxygen in electrolytes on the electrochemical properties of aqueous ZIBs is rare to date.

In this study, we demonstrate that the oxygen dissolved in aqueous ZnSO_4_ electrolytes indeed cause significant impact on the reversibility and cycling stability of Zn anode and ZIBs. For Zn/Zn symmetrical cell, after removing the oxygen from the electrolyte, the cycling life can be increased about 20 times than that in the presence of oxygen. For aqueous Zn-MnO_2_ battery, the presence of oxygen contributes an extra capacity over 20% to the cathode at the initial several cycles, but such capacity cannot be maintained in subsequent cycles. Instead, after removing the oxygen in advance, the decrease of capacity at the initial cycles is significantly suppressed, thus offering a more stable electrochemistry system. The impact of the dissolved oxygen on the cycling stability also exists in other aqueous ZIBs with cathodes of VO_2_, V_2_O_5_, Na_0.55_Mn_2_O_4_·1.5H_2_O and K_2_Zn_3_[Fe(CN)_6_]_2_·(H_2_O)_9_.

## Results

### Effect of Dissolved O_2_ on Zn Anode

**Figure 1:**
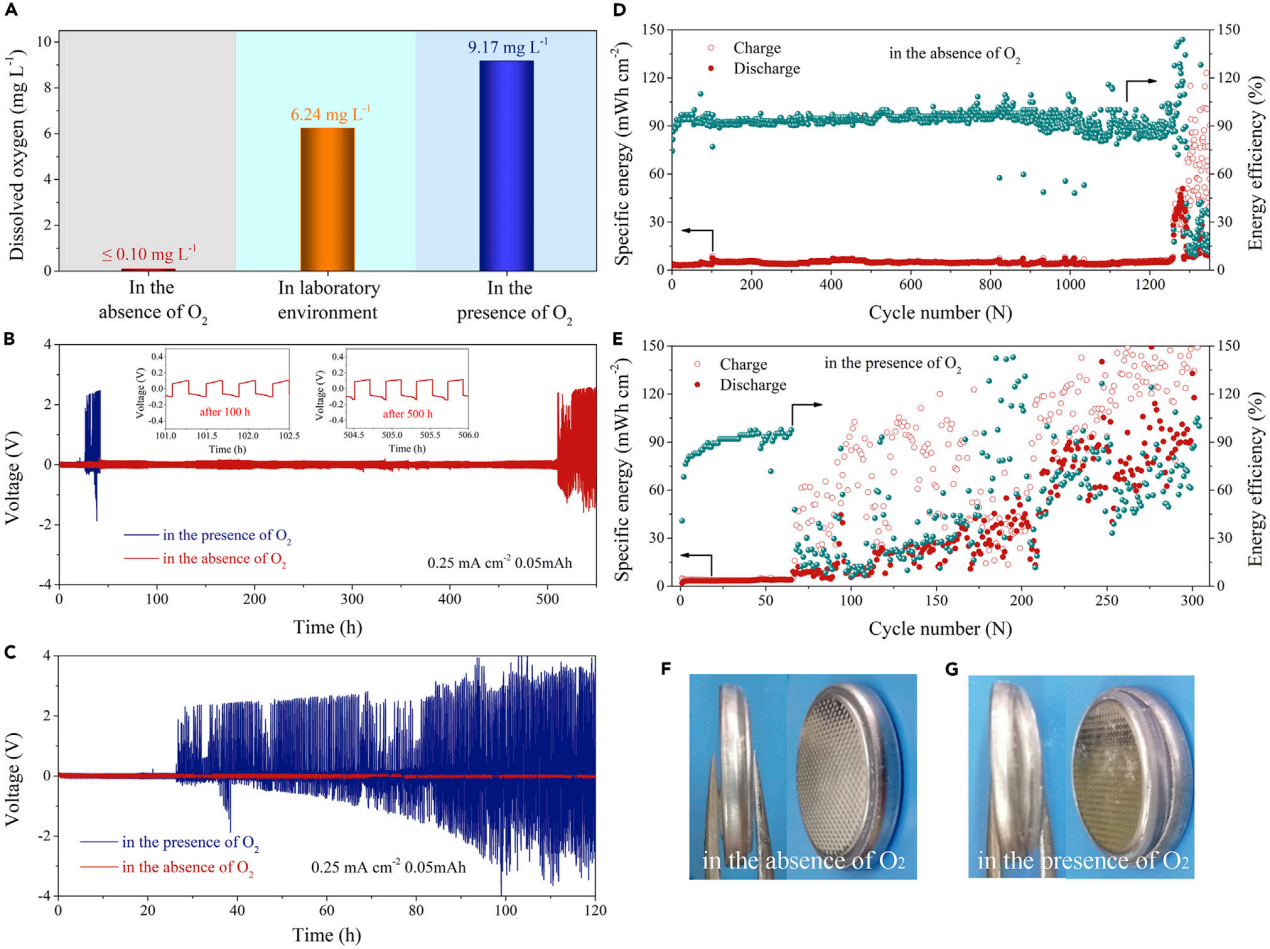
Electrochemical Characterization of Zn/Zn Cells (A) The contents of O_2_ in 2 M ZnSO_4_ aqueous electrolyte at three different conditions: in the absence of O_2_, in laboratory environment, and in the presence of O_2_. (B) Electrochemical stability and reversibility of the symmetrical Zn/Zn cell using 2 M ZnSO_4_ aqueous electrolyte in the presence of O_2_ and in the absence of O_2_, respectively, at a current density of 0.25 mA cm^−2^ and a charge/discharge cut-off capacity of 0.05 mAh. Insets are the galvanostatic charging-discharging curves of the Zn/Zn cell using the electrolyte in the absence of O_2_ after 100-h and 500-h cycling. (C) Electrochemical stability and reversibility of the symmetrical Zn/Zn cell in the presence of O_2_ and in absence of O_2_ with a cut-off cycling time of 120 h. (D–G) The specific energy and energy efficiency of the symmetrical Zn/Zn cells (D) in the absence of O_2_ and (E) in the presence of O_2_. Digital photographs of the Zn/Zn cells (F) in the absence of O_2_ and (G) in presence of O_2_ after the 120-hgalvanostatic cycling.

In order to study the impact of the dissolved O_2_ in ZnSO_4_ aqueous electrolyte on the reversibility and cycling stability of Zn anode and ZIB devices, we designed three electrolytes with different O_2_ conditions: in the absence of O_2_ (removing O_2_ using high-purity nitrogen), in laboratory environment (containing naturally dissolved O_2_), and in the presence of O_2_ (inletting with high-purity oxygen to reach O_2_ saturation). The corresponding dissolved O_2_ contents are listed in Figure 1A. The dissolved O_2_ in the 2 M ZnSO_4_ aqueous electrolyte reached 6.24 and 9.17 mg L^−1^ in the open laboratory environment and in the presence of O_2_ at room temperature, respectively. For comparison, the dissolved O_2_ content after removing O_2_ was reduced to less than 0.10 mg L^−1^.

The electrochemical stability of Zn anode in the presence of O_2_ and in absence of O_2_ was evaluated by long-term galvanostatic cycling of symmetrical Zn/Zn coin cells (Chao et al., 2018). As shown in Figure 1B, using the 2 M ZnSO_4_ aqueous electrolyte in the presence of O_2_, the Zn/Zn battery underwent a sudden and irreversible rise of the polarization voltage after cycling for 25 h at a current density of 0.25 mA cm^−2^. When using a low current density, the Zn^2+^ ions undergo a deep deposition and exfoliation on the electrode. The generation of side reactions due to oxygen and changes in the stability of the battery are more easily detected under such a low current density in the Zn/Zn symmetrical cell (Pan et al., 2016, Chao et al., 2019, Yang et al., 2019, Zhao et al., 2019b, Zhao et al., 2019a). In laboratory environment, the Zn/Zn cell also appeared distinct voltage polarization after 72 h (see Figure S1). As a contrast, as shown in Figure 1B, the Zn/Zn battery in the absence of O_2_ displayed the best stability and reversibility for Zn plating/stripping. Specifically, after eliminating O_2_, the cell exhibited a stable polarization voltage (~0.1 V) and a long-term galvanostatic cycling life of 510 h, representing a more than 20-fold improvement. The constant voltage curves after 100 and 500 h (insets of Figure 1B) also demonstrated the reversible Zn plating/stripping enabled in the absence of O_2_.

Energy efficiency presents the energy loss of a ZIB battery during charging and discharging processes (Chiasserini and Rao, 2000, Simunic et al., 2001, Zhang et al., 2011, Hu et al., 2013), and it can reflect the degree of deposition and exfoliation of Zn ions at a certain overpotential, thereby reflecting the stability of the battery. As shown in Figure 1D, the high-and-stable energy efficiency (~90% after 1,260 cycles) indicated the long-term cycling stability for Zn plating/stripping in the absence of O_2_. Comparatively speaking, the energy efficiency was unsatisfactory (less than 90%) after 65 and 180 cycles in the presence of O_2_ (Figure 1E) and in open laboratory environment (Figure S2), respectively. Furthermore, as shown in Figure 1C, the voltage was hold in the stable state (less than 0.1 V) of the entire cycling within cut-off cycling time of 120 h in the absence of O_2_. However, the voltage was significantly polarized after 26 h in the presence of O_2_. In addition, after a 120-h galvanostatic cycling, the Zn/Zn battery was no obvious volume swell in the absence of O_2_ (Figure 1F). For comparison, the battery showed obvious volume expansion in the presence of O_2_ (Figure 1G). This indicated that the dissolved O_2_ caused the thermodynamically instability of Zn and led to the hydrolysis of the electrolyte (Abdelall et al., 1992, Abdallah, 2003).

**Figure 2:**
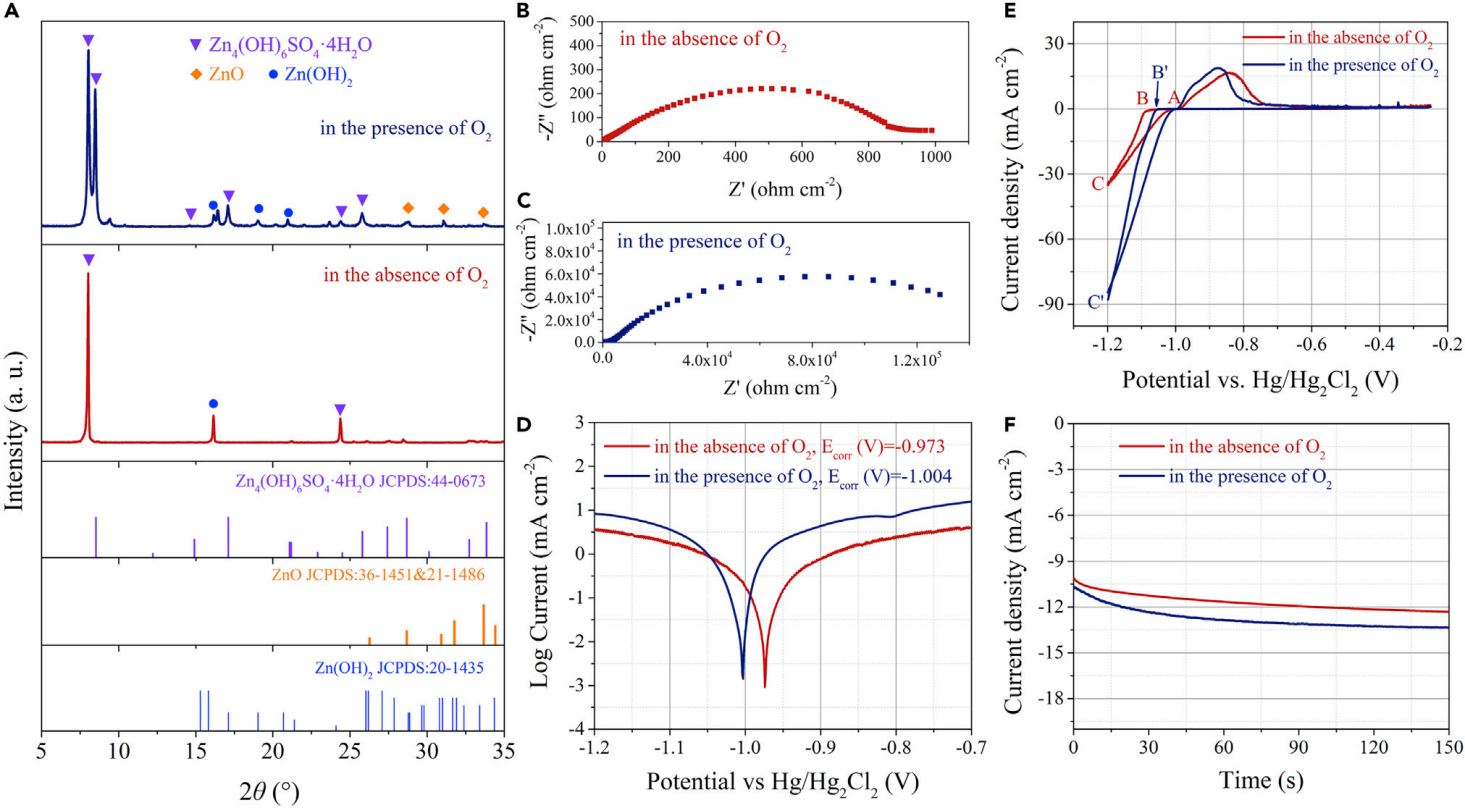
Effect of O_2_ on Zn (A–C) (A) X-ray diffraction (XRD) patterns of Zn after 120 h long-term galvanostatic cycling. Electrochemical impedance spectra (EIS) of symmetric Zn/Zn batteries in the absence of O_2_ (B) and in presence of O_2_ (C) after 120-h galvanostatic cycling. (D) Linear polarization curves showing the corrosion on Zn in the absence of O_2_ and in presence of O_2_. (E) The nucleation cyclic voltammograms (CVs) in the absence of O_2_ and in presence of O_2_. (F) Chronoamperograms (CAs) of Zn at a −150 mV overpotential in the absence of O_2_ and in presence of O_2_.

X-ray diffraction (XRD) characterization confirmed the corrosion of Zn after 120-h long-term galvanostatic cycling process. The pristine Zn showed no obvious zinc oxide, hydroxide, or zincate from its XRD pattern (JCPDS 87-0713, Figure S3), which is according with the result of scanning electron microscope (SEM) element mapping characterization (Figures S4A–S4C). For by-products on the surface of Zn plates, their peak positions were mainly concentrated within 5–35°. As shown in Figure 2A, there was a strong peak located at 8° for Zn plates both in the presence and absence of O_2_, which corresponds to Zn_4_(OH)_6_SO_4_·nH_2_O (Pan et al., 2016, Jin et al., 2019). However, in the presence of O_2_, there were more by-products (ZnO, Zn(OH)_2_, and zincate) on the surface of Zn plates. As for ZnO, the distinct peaks located at 28.7, 31.1, and 33.7°. Moreover, the shoulder peak located 8.5° was related to the complexation of ZnO with SO_4_^2−^ ions in the presence of O_2_. Zn(OH)_2_ on the Zn plate mainly concentrated at 16.1, 19.0, and 20.9° (Trócoli and La Mantia, 2015). Comparatively speaking, Zn plate in the absence of O_2_ did not show obvious ZnO signal, and there were only two diffraction peaks of Zn_4_(OH)_6_SO_4_·nH_2_O at 8 and 24.3° as well as a characteristic peak of Zn(OH)_2_ at 16.1°. This indicated that water and electrolyte ions can also cause slow corrosion on Zn plate in the absence of oxygen. Based on the above comparison, it can be verified that the Zn surface was severely corroded by O_2_ in the presence of O_2_ and formed a metal oxide/hydroxide layer, which caused electron insulation on the surface of Zn. Moreover, through the comparison of electrochemical impedance spectroscopies (EIS), it is shown that the presence of O_2_ accelerated the corrosion of Zn during electrochemical cycling (Figure S5). The impedance increased by 100 orders of magnitude in the presence of O_2_ (Figure 2C) than that in the absence of O_2_ (Figure 2B). In addition, the corrosion on the surface of Zn in the absence of O_2_ and in presence of O_2_ after long-term cycling process was further confirmed by SEM (Figures S4D–S4I and S6) and atomic force microscope (AFM, Figure S7 and Table S2) characterizations.

The effect of O_2_ on the Zn corrosion was further analyzed by linear polarization experiment in the 2 M ZnSO_4_ electrolyte (Figure 2D). Compared with the Zn in the presence of O_2_, the corrosion potential in the absence of O_2_ increased from −1.004 to −0.973 V, and the potential offset was 31 mV. Most notably, the corrosion current in the absence of O_2_ also reduced by 330 μA cm^−2^. The more positive corrosion potential and the lower corrosion current indicated a less tendency of corrosion reaction as well as a lower corrosion rate (Abdelall et al., 1992, Stupnisek-Lisac et al., 1995, Abdallah, 2003). Thus, the result from the linear polarization experiment proved that the dissolved O_2_ caused severe corrosion on the Zn plate.

As shown in Figure 2E, cyclic voltammetry (CV) measurements were carried out in a three-electrode configuration in which Zn plate is the counter electrode, Ti is the working electrode, and saturated calomel electrode (SCE) is the reference electrode. When sweeping the potential toward more positive values, the nucleation process showed a crossover characteristic. The potential point of *A* was the crossover potential, and the point of *B* (or *B′*) was the potential point of Zn^2+^ ions, which begins to be reduced. The potential offset between the crossover point (*A*) and the point (*B* or *B′*) was known as the overpotential of nucleation (NOP). The value of NOP was the reference to judge the extent of electrode polarization (Mackinnon et al., 1987, MacKinnon et al., 1990, Tripathy et al., 1997, Zhang and Hua, 2009). Compared with the NOP in the presence of O_2_, the NOP elevated by 29 mV (56–85 mV) in the absence of O_2_. The higher overpotential indicated the adequate force for the growth and nucleation process (Pei et al., 2017).

Furthermore, the application of a −150 mV overpotential to the chronoamperometry (CA) test was conducted, and the corresponding result is shown in Figure 2F. The ever-increasing current density indicated the rampant diffusion and rough deposition process (Diggle and Damjanovic, 1972, Trejo et al., 2001, Wang et al., 2001, Wang et al., 2006, Lan et al., 2007). Zn^2+^ ions more likely diffuse along the energetically favorable sites for charge transfer on the surface (Ballesteros et al., 2007, Zhao et al., 2019b, Zhao et al., 2019a). Moreover, Zn^2+^ ions tend to grow into dendrites for the sake of the minimum surface energy and the exposed area (Ballesteros et al., 2007). In this study, in the absence of O_2_, the initial Zn nucleation and 2D diffusion processes occurred within 20 s from the CA plots. Then, at a current density of 12 mA cm^−2^, the 3D diffusion processes become constant and stable, which indicated the appeared of constrained 2D surface diffused of the locally Zn^0^. This was because Zn^2+^ ions were tended to be uniformly deposited on the 2D plane in an O_2_-free environment. In the presence of O_2_, during the initial nucleation process, because Zn^2+^ ions were tended to grow toward the lowest surface energy, the deposition was heterogeneous and unstable.

Through the above analyses, we conclude that the O_2_ dissolved in ZnSO_4_ aqueous electrolyte accelerates the corrosion on the Zn surface and generates by-products such as zinc oxide, zinc hydroxide, and zincate. The by-products with insulation property irreversibly increase the electrochemical impedance of Zn, thereby resulting in the instability of symmetric Zn/Zn battery.

## Discussion

### Effect of Dissolved O_2_ on Zn-MnO_2_ Battery

In order to study the impact of the dissolved O_2_ on ZIB devices, we constructed Zn-MnO_2_ batteries using α-MnO_2_ nanofibers as cathode material that were synthesized by hydrothermal method. Figures S8 and S9 show the microstructure of α-MnO_2_ nanofibers. Firstly, an aqueous Zn-MnO_2_ battery was assembled using 2 M ZnSO_4_ electrolyte in the open laboratory environment (the content of dissolved O_2_ was 6.24 mg L^−1^). As shown in Figure S10A, although a high reversible capacity was delivered in the first cycle (173 mAh g^−1^ at a current density of 0.308 A g^−1^), a rapid capacity deterioration was observed upon cycling. The specific capacity was decreased from 173 to 21 mAh g^−1^ after 600 cycles, and the capacity retention rate was only 12.1%. Especially in the initial tens of cycles, the capacity showed cliff-type decay. This capacity fading was explained because of the dissolution of Mn^2+^ from Mn^3+^ disproportionation into the electrolyte and the formation of Zn dendrite during cycling (Lee et al., 2014, Alfaruqi et al., 2015a, Alfaruqi et al., 2015b, Boeun et al., 2015, Mathew et al., 2015).

**Figure 3:**
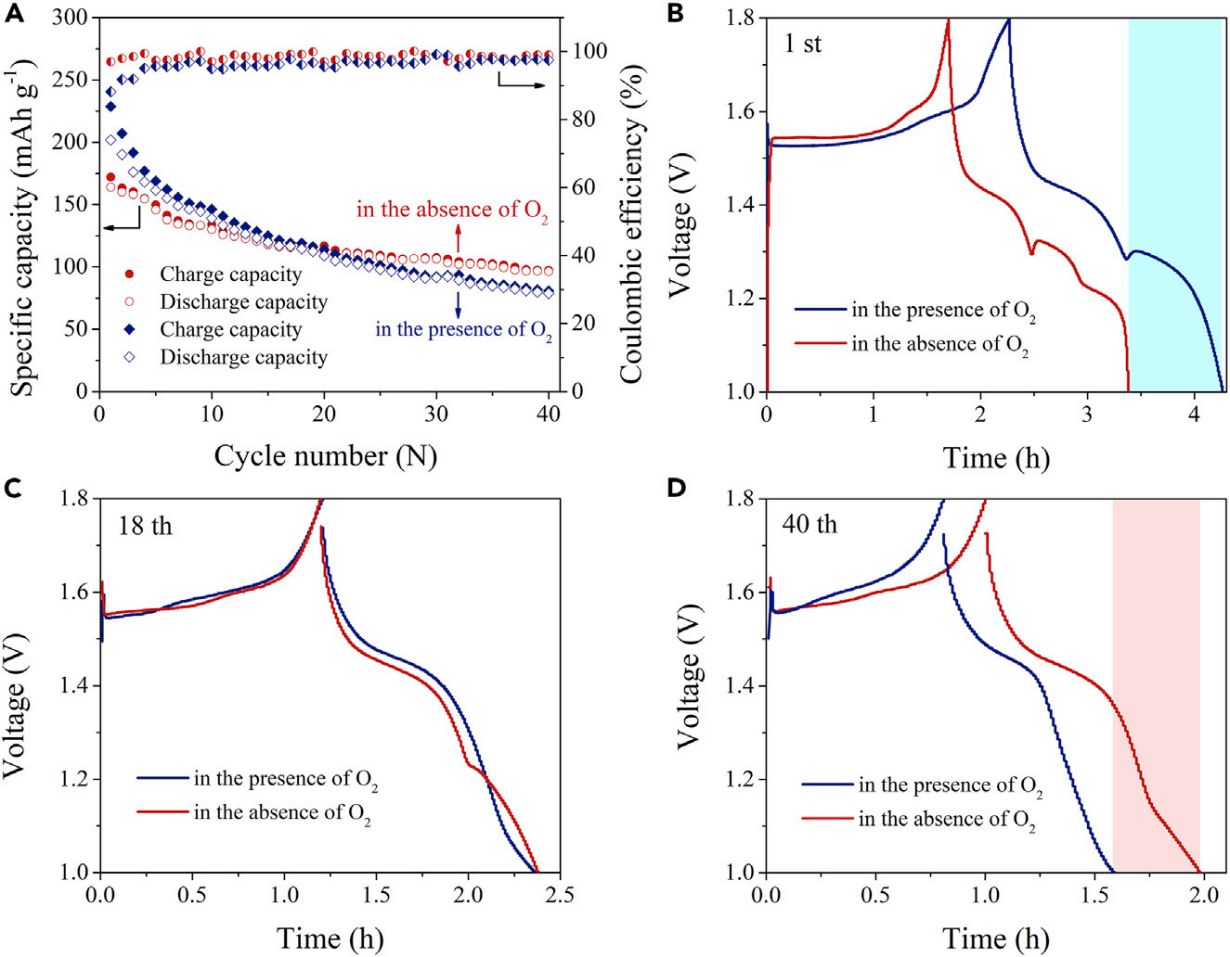
Electrochemical Characterization of Aqueous Zn-MnO_2_ Batteries (A) Cycling stability at 0.1 A g^−1^ in the presence of O_2_ and in absence of O_2_. (B–D) Galvanostatic charge and discharge (GCD) curves at different cycles: (B) the first cycle, (C) the 18^th^ cycle, and (D) the 40^th^ cycle.

Following, aqueous Zn-MnO_2_ batteries were assembled using 2 M ZnSO_4_ electrolytes in the presence of O_2_ and in absence of O_2_, respectively, and the comparison of their cycling performance is shown in Figure 3A. The initial capacity of the aqueous Zn-MnO_2_ battery in the absence of O_2_ was less than that in the presence of O_2_ (164 mAh g^−1^ compared with 202 mAh g^−1^ at 0.1 A g^−1^). Here, the α-MnO_2_ used in Zn-MnO_2_ batteries has been widely reported in some high-level literatures, and all the reported works showed that the depth of charge/discharge was approximate with that employed in our work (Pan et al., 2016, Chen et al., 2017, Fang et al., 2018, Song et al., 2018, Zheng et al., 2019). We compared the cycling stability of aqueous Zn/MnO_2_ batteries using 2 M ZnSO_4_ electrolytes in the presence of O_2_ and in absence of O_2_ at 0.1 A g^−1^ without adding conductive graphite of cathode material (Figure S11). A high reversible capacity was delivered in the first cycle in the presence of O_2_. Thus, the extra charge capacity was not due to the oxidation of conductive graphite. Nevertheless, the battery showed a better cycling stability in the absence of O_2_ (60% of capacity retention) than that in the presence of O_2_ (38% of capacity retention) after 40 cycles. Galvanostatic charge and discharge (GCD) curves also showed the aqueous Zn-MnO_2_ battery in the presence of O_2_ exhibited a higher capacity within the initial cycles than that in the absence of O_2_ (Figure 3B), and the former had an obvious discharge platform at the third stage. But after the rapid capacity attenuation, the capacity in the presence of O_2_ was approximate with that in the absence of O_2_ at 18 cycles (Figure 3C). After 40 cycles, its capacity was obviously lower than that in the absence of O_2_ (Figure 3D), and the third discharge platform disappeared as well. Such reaction process may correspond to the ZnSO_4_[Zn(OH)_2_]_3_·*x*H_2_O discharge product. The presence of ZnSO_4_[Zn(OH)_2_]_3_·*x*H_2_O indicated the H^+^ conversion reaction with MnO_2_ cathode to form MnOOH during the third discharge platform. Meanwhile, the OH^−^ reacted with the Zn^2+^ ions, which dissolved from Zn anode and ZnSO_4_ aqueous electrolyte to form the accompanied discharge compound of ZnSO_4_[Zn(OH)_2_]_3_·*x*H_2_O on MnO_2_ electrode along with the redox reaction of MnO_2_+H^+^+e^−^↔MnOOH (Pan et al., 2016, Jin et al., 2019). In addition, the Zn anodes of two batteries were subjected to SEM characterization after cycling (Figure S12). It was observed that the Zn anode was severely corroded in the cycled battery using the electrolyte with O_2_. Similarly, when widening the voltage window to 1–1.9 V, the corresponding Zn-MnO_2_ battery exhibited a faster capacity attenuation in the O_2_-rich environment (Figure S10B). The results proved that the existence of dissolved O_2_ indeed resulted in the corrosion of Zn anode and thus aggravated the capacity decay of the Zn-MnO_2_ battery. We conclude other might reasons that caused the rapid capacity fading of Zn-ion batteries. The insertion of proton and hydrogen evolution during recharge is the reason for the rapid capacity fading—the proton produced from the ZnSO_4_ electrolyte and the hydrolysis of water (H_2_O ↔ H^+^+OH^−^). The growth of notorious Zn dendrites as well as their poor electrochemical and thermodynamic characteristics have been the bottleneck that restrict Zn-ion batteries for long cycling stability. More importantly, because of incessant complex Faraday and non-Faraday side reactions in ZIBs, the Zn^2+^-insulating by-products, such as Zn oxides, hydroxides, and zincates, can passivate the fresh Zn. In addition, the dissolution of electrode materials into the bulk electrolyte is another reason for the capacity fading in aqueous Zn-ion batteries (Pan et al., 2016, Fang et al., 2018, Song et al., 2018).

### O_2_ Dissolved in Electrolyte on the Energy Storage Electrochemistry

**Figure 4:**
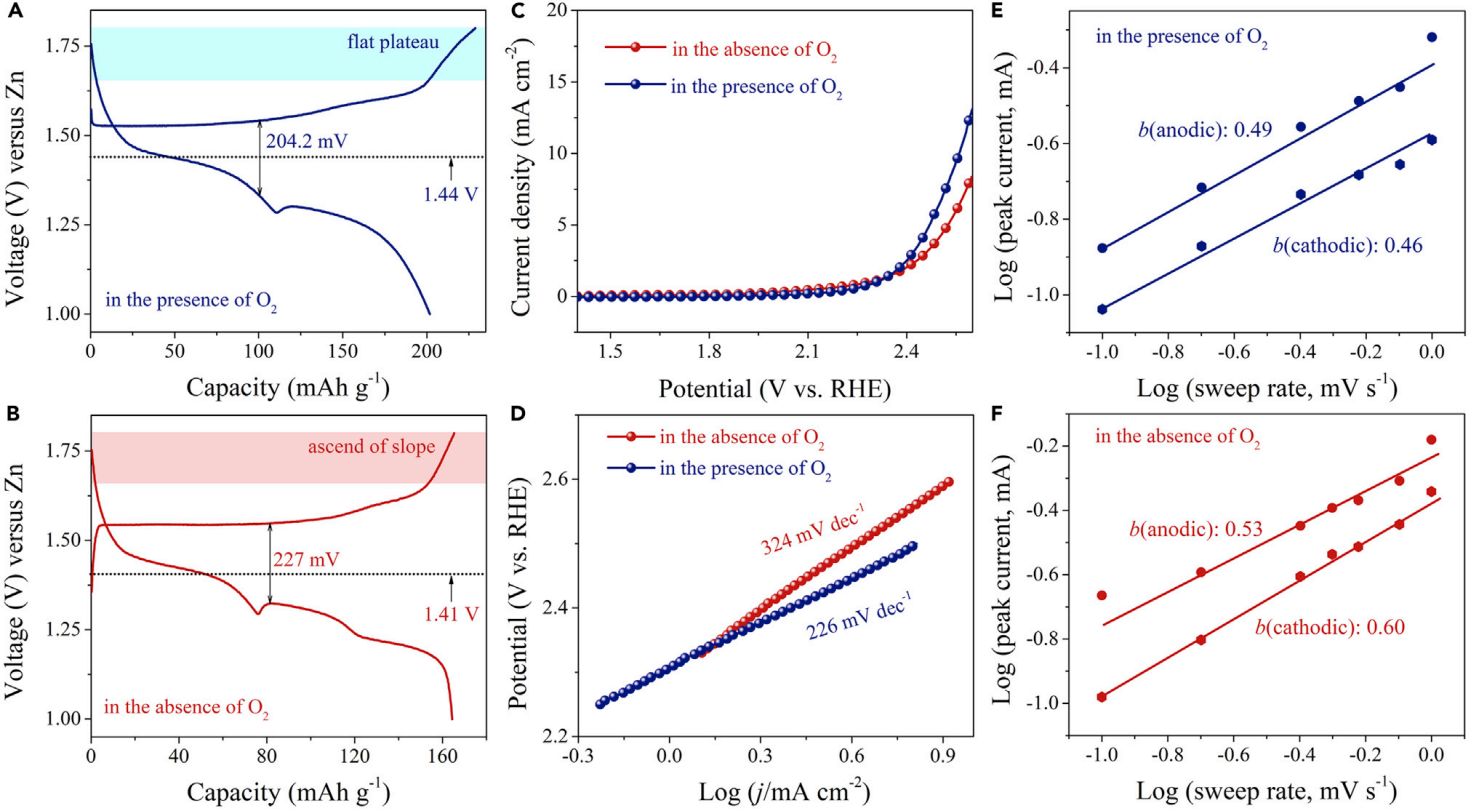
The Effect of O_2_ on the Electrochemistry of Zn-MnO_2_ Battery The first GCD curves of Zn-MnO_2_ batteries (A) in the presence of O_2_ and (B) in absence of O_2_ at 0.1 A g^−1^. Linear sweep voltammetry (LSV) curves (C) and the corresponding Tafel plots (D) of α-MnO_2_ electrodes under the three-electrode system in the presence of O_2_ and in absence of O_2_. The determination of the *b* values of Zn-MnO_2_ batteries under the conditions (E) in the presence of O_2_ and (F) in absence of O_2_.

For a deeper understanding of the effect of O_2_ dissolved in electrolyte on the energy storage electrochemistry of Zn-MnO_2_ battery, we investigated the influence of O_2_ on the electrochemical properties of α-MnO_2_ cathode, including the GCD, linear sweep voltammetry (LSV), and *b* values. In ambient of O_2_, the initial charge voltage profile exhibited a flat plateau at 1.52 V, and then a voltage plateau from 1.65 to 1.8 V was observed (Figure 4A). It was evidently different from that occurred in the absence of O_2_ (Figure 4B), where the charge voltage profile showed a flat plateau at 1.54 V, and then the slope of voltage curve ascended without obvious plateau from 1.65 to 1.8 V. The average operating voltages of α-MnO_2_ were 1.44 V (in the presence of O_2_) and 1.41 V (in the absence of O_2_), and the nontrivial overpotentials were 204.2 mV (in the presence of O_2_) and 227 mV (in the absence of O_2_) at the first cycle. Moreover, the battery in the presence of O_2_ contributed more capacity (over 20%) than that in absence of O_2_ at the first cycle.

As shown in Figure 4C, α-MnO_2_ electrode exhibited a smaller overpotential (58 mV) at 5 mA cm^−2^ in the presence of O_2_ than that in the absence of O_2_. LSV characterization indicated that, although the oxygen evolution reaction (OER) catalytic activity of the α-MnO_2_ was unsatisfactory when compared with some typically reported OER catalysts(Ma et al., 2018, Pendashteh et al., 2019, Shinde et al., 2019), the hydrolysis was easier in the O_2_-rich environment. Subsequently, the corresponding Tafel slopes were analyzed to show the OER catalytic kinetics (Ma et al., 2018). As shown in Figure 4D, α-MnO_2_ exhibited a Tafel slope of 226 mV dec^−1^ in the presence of O_2_, lower than that in the absence of O_2_ (324 mV dec^−1^), which suggested the improved OER kinetics performance of the α-MnO_2_ electrode in the presence of O_2_. Moreover, the *b* value of the Zn-MnO_2_ battery in the presence O_2_ (Figure 4E) demonstrated a more verge on capacity contribution from battery-type energy storage than that in the absence of O_2_ (Figure 4F). In addition, the CV curves (Figures S13A and S13B) showed that the oxidation peak threshold position was 1.67 V in the presence of O_2_ at 0.2 mV s^−1^, which appeared later than that in the absence of O_2_ (1.63 V). Thus, O_2_ participated in the capacity contribution of the α-MnO_2_ cathode. The overall reactions for Zn-MnO_2_ battery in ZnSO_4_ electrolyte (in the presence of O_2_) can be summarized as the following process during charging and discharging process: MnO_2_ + *1/4*O_2_ + Zn + *(15+4x)/12*H_2_O + *1/3*ZnSO_4_↔MnOOH + *1/3*ZnSO_4_[Zn(OH)_2_]_3_·*x*H_2_O. The formation of MnOOH indicates a possible, alternative conversion reaction of Zn^2+^ ion intercalation into MnO_2_. It is likely that MnO_2_ reacts with a proton from water to form MnOOH (MnO_2_+H^+^+e^−^+ ↔MnOOH). The reaction mechanism is the active conversion reaction between MnO_2_ and H^+^. As a result, we can draw a conclusion that the combination of Zn anode and α-MnO_2_ cathode in 2 M aqueous ZnSO_4_ electrolyte without oxygen presents a high-reversibility and high-cycling-stability aqueous Zn-MnO_2_ battery.

### Effect of the Dissolved O_2_ Exists in Other Types of Aqueous ZIBs

**Figure 5:**
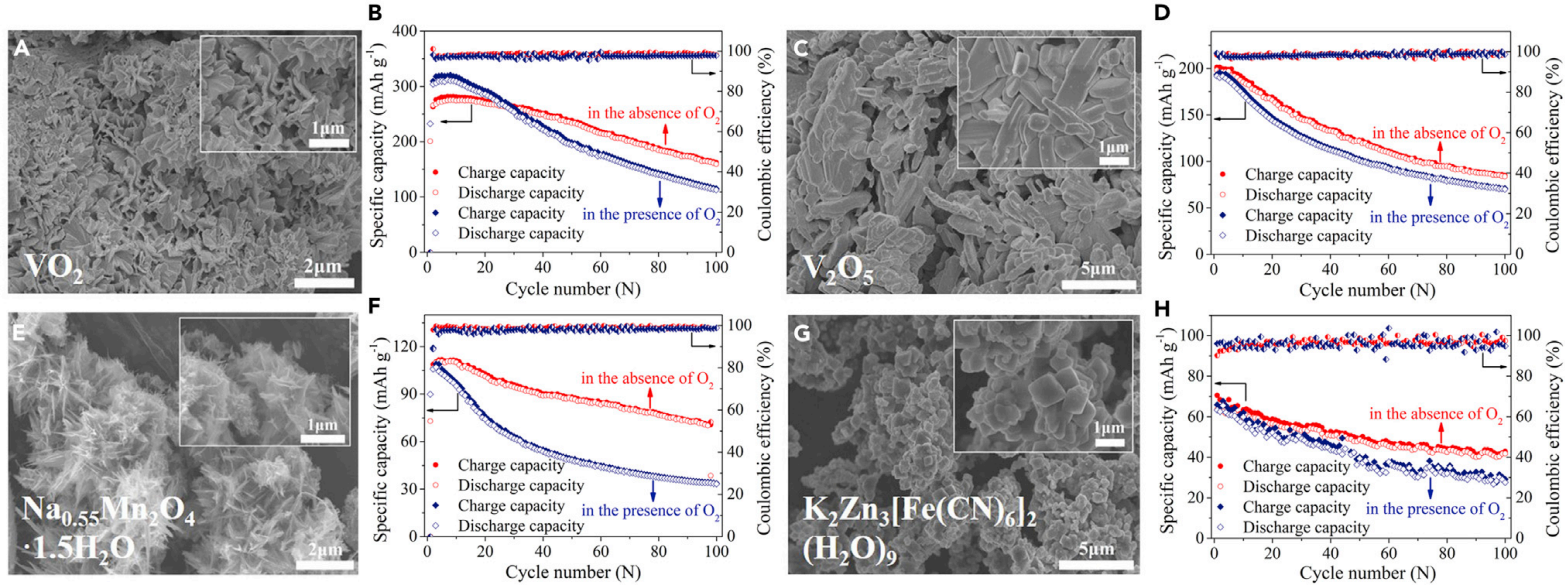
The Impact of the Dissolved O_2_ in Aqueous ZIBs with Cathodes of VO_2_, V_2_O_5_, Na_0.55_Mn_2_O_4_·1.5H_2_O, and K_2_Zn_3_[Fe(CN)_6_]_2_·(H_2_O)_9_ Using the 2 M ZnSO_4_Electrolytes in the Presence of O_2_ and in Absence of O_2_ (A) SEM characterization of VO_2_. (B) Cycling stability of Zn-VO_2_ batteries at 0.1 A g^−1^ with a voltage window of 0.2–1.6 V. (C) SEM characterization of V_2_O_5_. (D) Cycling stability of Zn-V_2_O_5_ batteries at 0.1 A g^−1^ with a voltage window of 0.2–1.6 V. (E) SEM characterization of Na_0.55_Mn_2_O_4_·1.5H_2_O. (F) Cycling stability of Zn-Na_0.55_Mn_2_O_4_·1.5H_2_O batteries at 0.1 A g^−1^ with a voltage window of 1–1.8 V. (G) SEM characterization of K_2_Zn_3_[Fe(CN)_6_]_2_·(H_2_O)_9_. (H) Cycling stability of Zn-K_2_Zn_3_[Fe(CN)_6_]_2_·(H_2_O)_9_ batteries at 0.1 A g^−1^ with a voltage window of 0.8–1.9 V.

To prove whether the impact of the dissolved O_2_ exists in other types of aqueous ZIBs, we constructed other four ZIBs using home-made VO_2_, V_2_O_5_, Na_0.55_Mn_2_O_4_·1.5H_2_O, and K_2_Zn_3_[Fe(CN)_6_]_2_·(H_2_O)_9_ as cathodes and using 2 M ZnSO_4_ solutions in the presence of O_2_ and in absence of O_2_ as electrolytes. Figures S14–S17, 5A, 5C, 5E, and 5G show the microstructures of the four cathode materials. Importantly, as shown in Figures 5B, 5D, 5F, and 5H, all of the ZIBs showed better cycling stability in the O_2_-free environment than those in the presence of O_2_. It indicated that such impact of the dissolved oxygen on the cycling stability was general in different types of ZIBs. It should be noted that, among these four ZIBs, Zn-VO_2_ battery in the presence of oxygen showed a higher initial capacity than that in the absence of oxygen, which was similar to Zn-MnO_2_ battery. It might be attributed to the electrocatalytic activity of VO_2_ with oxygen (Wan et al., 2019).

In summary, we studied the electrochemical reversibility of Zn anode and aqueous Zn-MnO_2_ battery with the consideration of O_2_ dissolved in electrolyte. Zn anode will react with O_2_ during cycling process, resulting in the instability of Zn/Zn symmetrical battery. By eliminating O_2_ in the electrolyte, the cycling life of Zn/Zn battery can increase more than 20 times. For Zn-MnO_2_ battery, the removal of O_2_ from the electrolyte can eliminate the oxygen corrosion on Zn anode, thus offering a better energy storage system with a higher capacity retention during cycling. The impact of the dissolved oxygen on the cycling stability also exists in other ZIBs using different cathodes, including VO_2_, V_2_O_5_, Na_0.55_Mn_2_O_4_·1.5H_2_O, and K_2_Zn_3_[Fe(CN)_6_]_2_·(H_2_O)_9_. The study not only is conducive to understand the electrochemistry of aqueous ZIBs more accurately but also provides useful information to design better aqueous ZIBs by controlling oxygen dissolved in electrolytes.

### Limitations of the Study

We revealed the impact of oxygen dissolved in electrolytes on aqueous zinc-ion batteries here. The study may need more rigorous analyses and evidence (such as *in-situ* or *in-operando* experiments) for the reaction mechanism of oxygen on zinc-ion batteries.

## Methods

All methods can be found in the accompanying Transparent Methods supplemental file.

## Data and Code Availability

The data that support the findings of this study are available from the corresponding author upon reasonable request.

## Supplemental Information

Document S1. Transparent Methods, Figures S1–S17, and Tables S1 and S2
