# Supplementary material for: Revealing the Impact of Oxygen Dissolved in Electrolytes on Aqueous Zinc-Ion Batteries
Source: iScience. 2020 Mar 20;23(4):100995. doi: 10.1016/j.isci.2020.100995 (PMC7132168; doi:10.1016/j.isci.2020.100995)
Supplement: Document S1. Transparent Methods, Figures S1–S17, and Tables S1 and S2 [file mmc1.pdf]

iScience, Volume *23*

Supplemental Information

Revealing the Impact of Oxygen Dissolved in Electrolytes on Aqueous Zinc-Ion Batteries

Lijun Su, Lingyang Liu, Bao Liu, Jianing Meng, and Xingbin Yan

Transparent Methods

Material Synthesis

α-MnO_2 was synthesized by a typical method,(Pan et al., 2016) 2 mL 0.5 M H_2SO_4 and 0.003 M $MnSO_4·H_2O$ were added to 90 mL deionized water. And 20 mL 0.1 M $KMnO_4$ was slowly added into the above mixed solution. The mixture was magnetically stirred for 2 h. Then, the mixed solution was transferred to a Teflon-lined autoclave and heated at 120 °C for 12 h. The obtained products were collected by centrifugation, washed three times with water, and dried by a vacuum oven at room temperature.

The $Na_{0.55}Mn_2O_4·1.5H_2O$ were synthesized by the molten salts method.(Hu et al., 2017) 5 g $NaNO_3$ was added into a crucible and then transferred to the muffle furnace at a temperature of 350 °C for 10 minutes. After $NaNO_3$ became molten state completely, 0.2 g $MnSO_4$ was added into the molten salt and heated for 1 minute. Then, the reaction product was taken out from the muffle furnace and cooled to room temperature. The final product was washed by deionized water and dried at room temperature.

V_2O_5 was synthesized as follows:(Zhang et al., 2018) commercial graphite and commercial V_2O_5 powder with a mass ratio of 2:8 were added into a hard steel ball-milled jar. The mixture was ball milled in a ball miller at 400 rpm for 180 minutes.

VO_2 was synthesized by a typical fabrication,(Ding et al., 2018) 1.8 g $H_2C_2O_4·2H_2O$ and 1.2 g V_2O_5 were added into 40 mL deionized water, and the above mixed solution was reacted at 75 °C for 60 minutes. Then, the above dispersion was transferred into a Teflon-lined autoclave and heated at 180 °C for 180 minutes. Finally, the product was collected and washed with ethanol and deionized water and frozen-drying for 12 h.

$K_2Zn_3[Fe(CN)_6]_2{\cdot}(H_2O)_9$ was synthesized via the following method:(Zhang et al., 2015) 100 mL 0.05 M $K_3Fe(CN)_6$ and 100 mL 0.1 M $ZnSO_4$ were added into 50 mL H_2O under vigorous stirring at 60 °C. After reaction, the suspensions were left to stand for several hours. Finally, the obtained powder was filtered, washed with deionized water, then dried at 70 °C.

Characterizations

The crystalline structure of the as-obtained material was characterized by powder X-ray diffraction pattern (XRD, Rigaku D/Max-2400, Cu-Kα radiation, λ = 0.15405 nm). Surface morphology and microstructure were investigated through field emission scanning electron microscope (FESEM, JSM 6701F), transmission electron microscope (TEM, JEOL 2100 FEG), high-resolution TEM (HRTEM, JOEL, JEM-2010 F) and atomic force microscope (AFM, MultiMode 8).

Electrochemical measurements

Oxygen evolution reaction (OER) test was performed in three-electrode system under the program of linear sweep voltammetry (LSV) with a scan rate of 5 mV s^{-1} and a voltage window of 0 to 1.8 V. The glassy carbon electrode was used as the working electrode, the saturated calomel electrode as the reference electrode and a platinum electrode as the counter electrode.

Corrosion test and cyclic voltammetry (CV) were carried on an electrochemical workstation (CHI660D, Chenhua, Shanghai, China). Linear polarization and chronoamperograms (CAs) measurements were conducted in a three-electrode configuration, in which bare Zn was used as the working electrode, Zn plate as the

counter, and saturated calomel (SCE) as the reference electrodes, respectively. The corrosion potential and corrosion current were calculated from Tafel plots. Electrochemical impedance spectrum (EIS) were recorded on the electrochemical workstation with a frequency range from 1 MHz to 0.01 Hz. Galvanostatic charge-discharge (GCD) measurements were performed with a battery test system (Land CT2001A model, Wuhan Land Electronics Ltd.). And the energy efficiency data of the symmetrical Zn/Zn battery was obtained directly from LAND system.

Fabrication of coin cells: The positive electrodes comprised active material powder (80 wt%), conductive graphite (10 wt%) and carboxymethylcellulose (CMC) (10 wt%). The active material could be the one of α-MnO_2, $Na_{0.55}Mn_2O_4\cdot1.5H_2O$, VO_2, V_2O_5 or $K_2Zn_3[Fe(CN)_6]_2\cdot(H_2O)_9$. The mass loading for the cathode materials was about 1 mg cm^{-2} on the stainless-steel grid current collector. The anode electrodes were Zn plates of 1×1 cm^{-2}. Each battery was assembled into a coin cell with a glass fiber as the separator and the 2 M $ZnSO_4$ as the aqueous electrolyte. Specifically, coin cells were assembled in different atmosphere: in an open laboratory atmosphere (dissolved O_2 content was 6.24 mg L^{-1}), in the presence of O_2 (dissolved O_2 content was 9.17 mg L^{-1}) and in the absence of O_2 (dissolved O_2 content was less than 0.1 mg L^{-1}).

Supplemental Figures

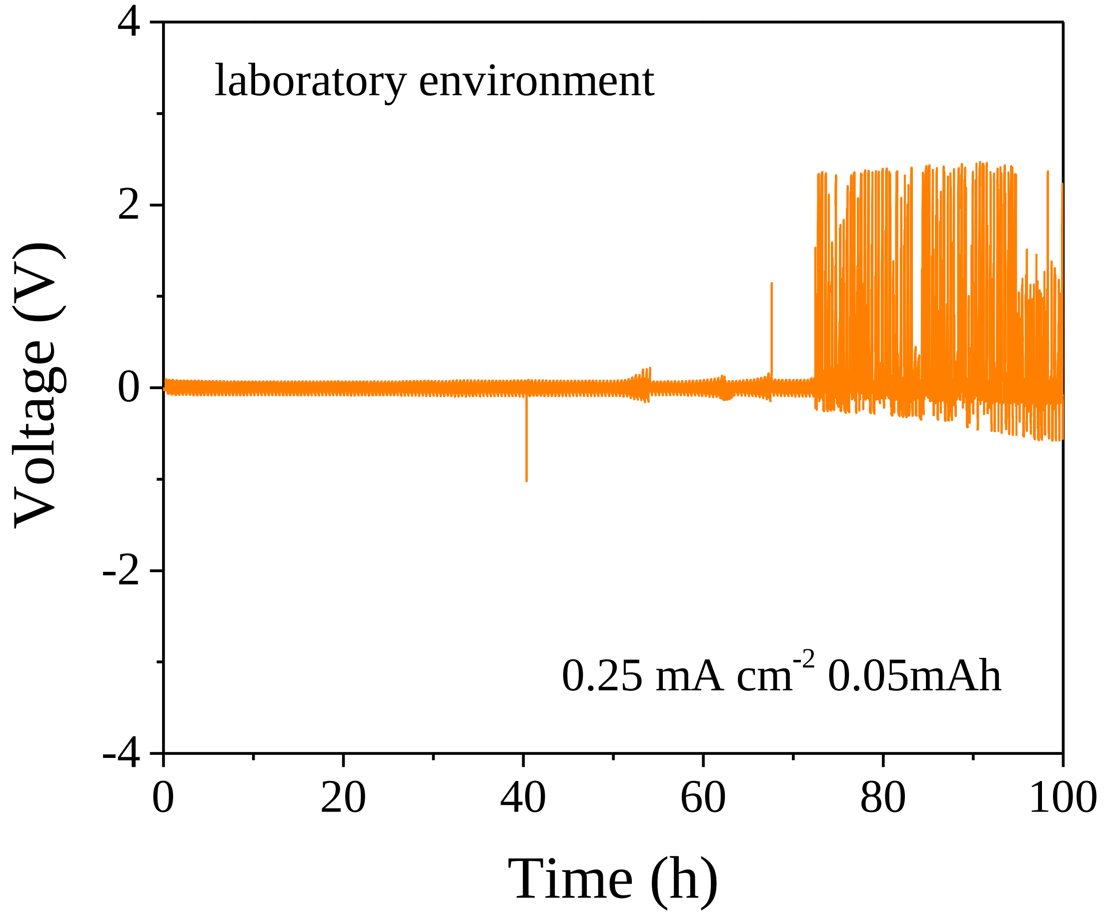

Figure S1. Characterization of Zn/Zn cell, related to Figure 1.

Electrochemical stability and reversibility of the symmetrical Zn/Zn cell using 2 M $ZnSO_4$ aqueous electrolyte in open laboratory environment at a current density of 0.25 mA cm^{-2} and a charge/discharge cut-off capacity of 0.05 mAh.

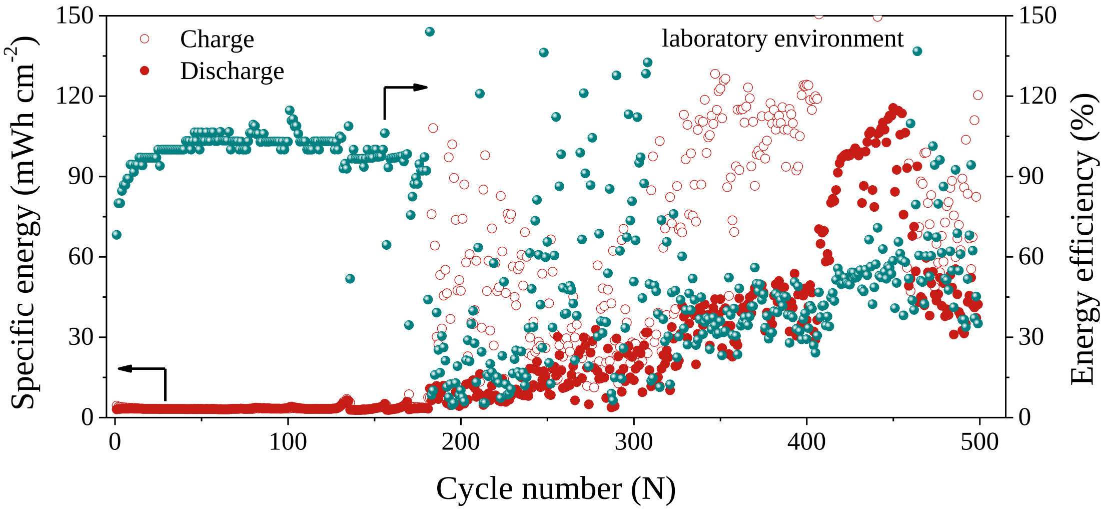

Figure S2. Characterization of Zn/Zn cell, related to Figure 1.

The specific energy and energy efficiency of the symmetrical Zn/Zn cell in open laboratory environment.

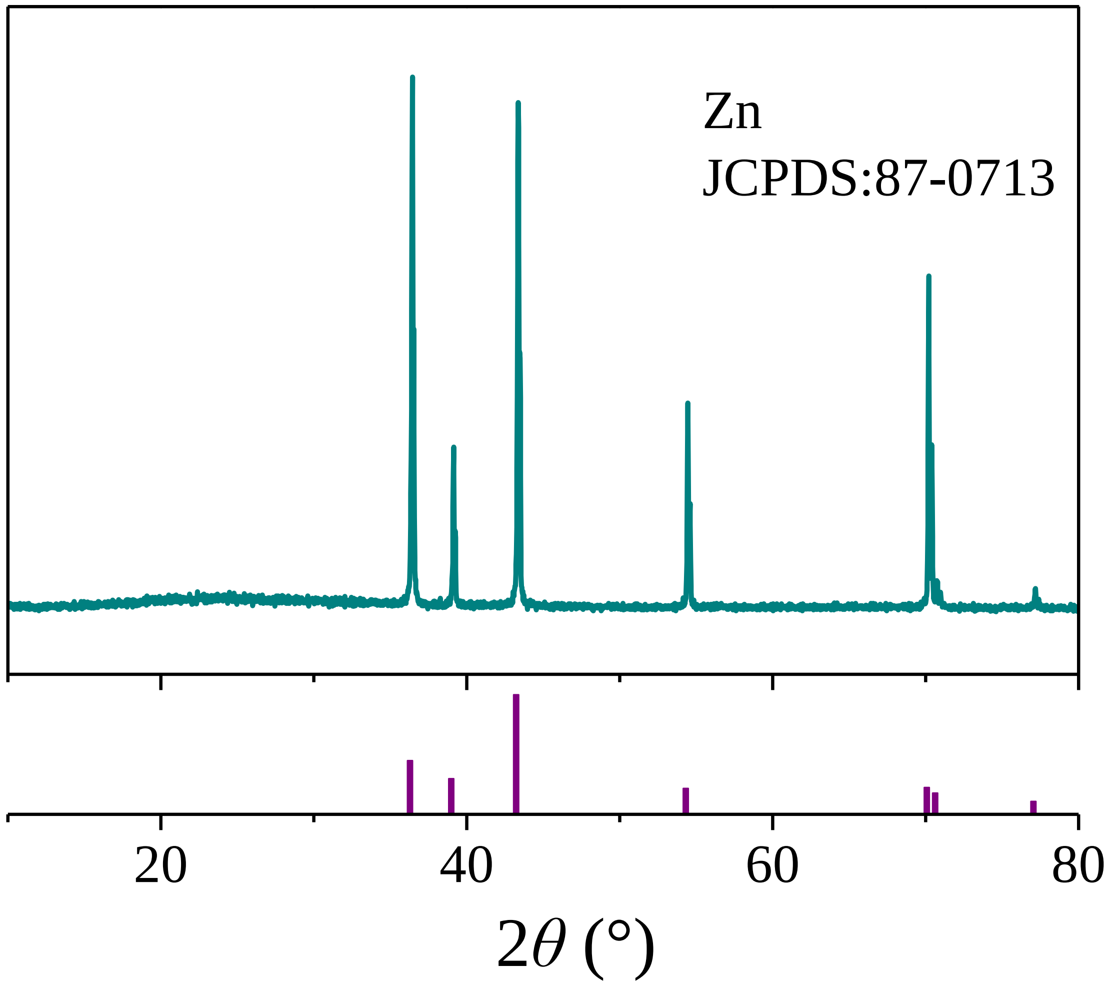

Figure S3. Characterization of Zn, related to Figure 2.

XRD pattern of pristine Zn plate.

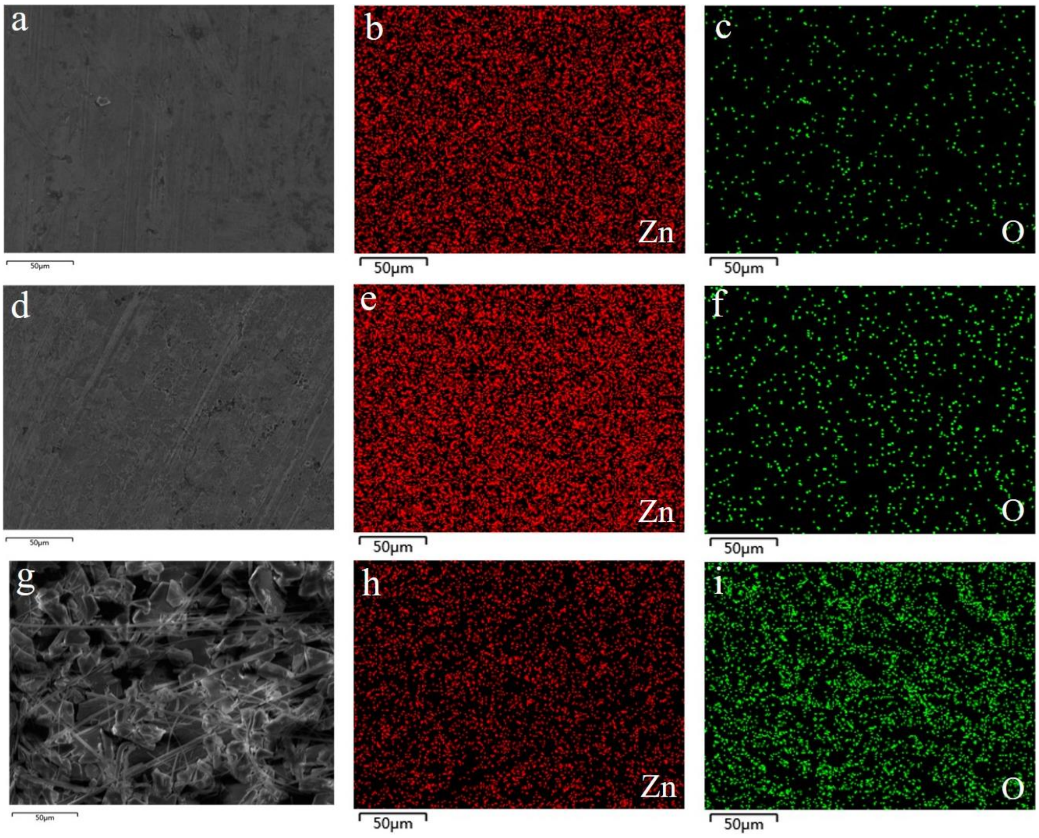

Figure S4. Characterization of Zn, related to Figure 2.

SEM as well as element mapping images of Zn. (a-c) Pristine Zn plate, (d-f) Zn plate after galvanostatic cycling for 120 h in the absence of O_2, (g-i) Zn plate after galvanostatic cycling for 120 h in the presence of O_2.

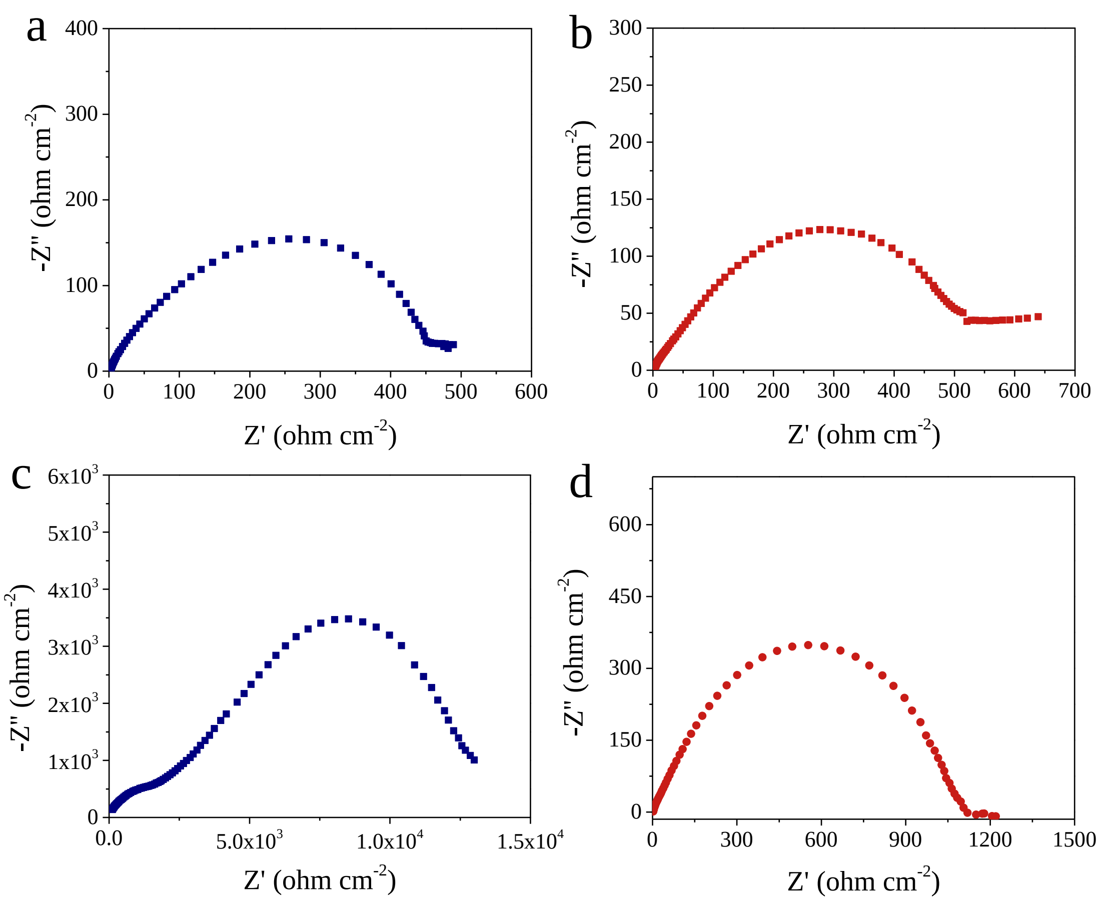

Figure S5. Impedance study of Zn/Zn cells, related to Figure 2.

EIS plots of symmetric Zn/Zn cells. EIS plots of pristine symmetric Zn/Zn cells in the presence of O_2 (a) and in absence of O_2 (b). (c) EIS plot of symmetric Zn/Zn cell after galvanostatic cycling for 30 h in the presence of O_2. (d) EIS plot of symmetric Zn/Zn cell after galvanostatic cycling for 500 h in the absence of O_2.

EIS plots showed that the initial symmetric Zn/Zn batteries assembled in the presence of O_2 (Figure S5a) and in absence of O_2 (Figure S5b) exhibited small electrochemical impedance. In the presence of O_2, the impedance of the symmetric Zn/Zn battery significantly increased by 100 orders of magnitude after galvanostatic cycling for 30 h (Figure S5c). It indicated that the surface of Zn was corroded, which caused electron insulation on the surface of Zn. When the dissolved O_2 was removed, the internal

corrosion of the battery was greatly reduced. After cycling for 120 h (Figure 2b), the electrochemical impedance increased slightly compared with that of the initial symmetric Zn/Zn battery before cycling, indicating the internal stability of the battery. As shown in Figure S5d, after cycling 500 h, the electrochemical impedance slightly increased due to the slow corrosion of Zn.

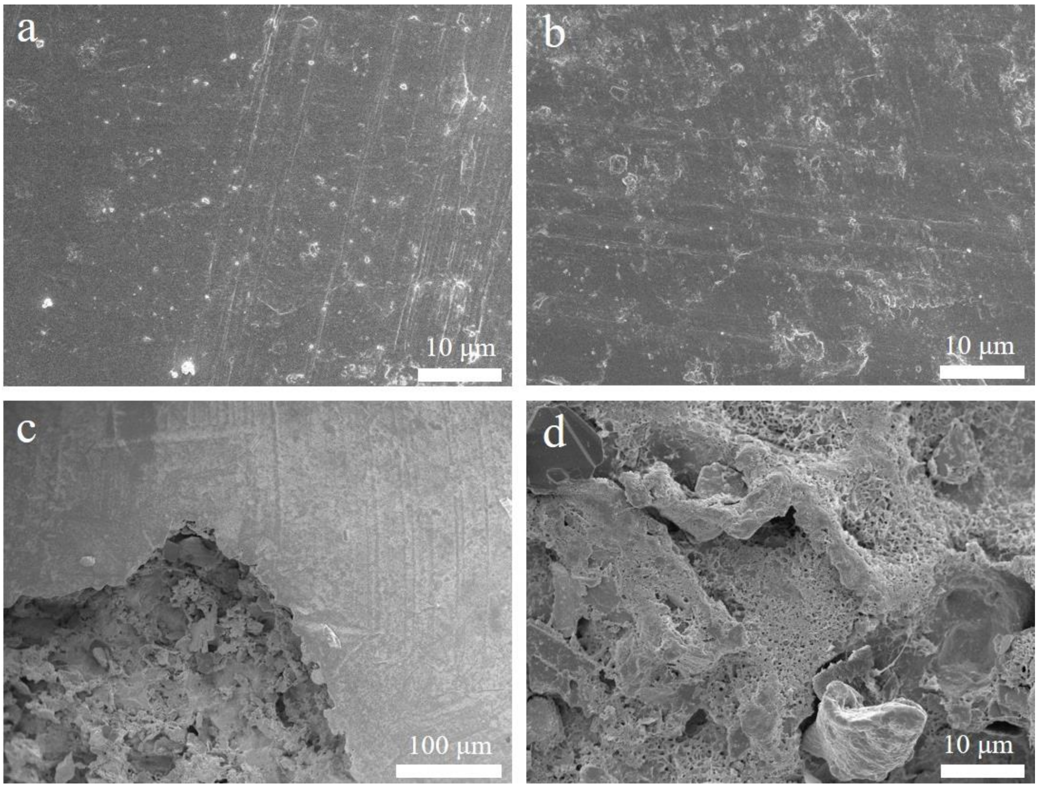

Figure S6. Morphologies of Zn, related to Figure 2.

SEM images of Zn plate. (a) Pristine Zn plate. Zn plates after galvanostatic cycling for 120 h (b) in the absence of O_2 and (c, d) in presence of O_2.

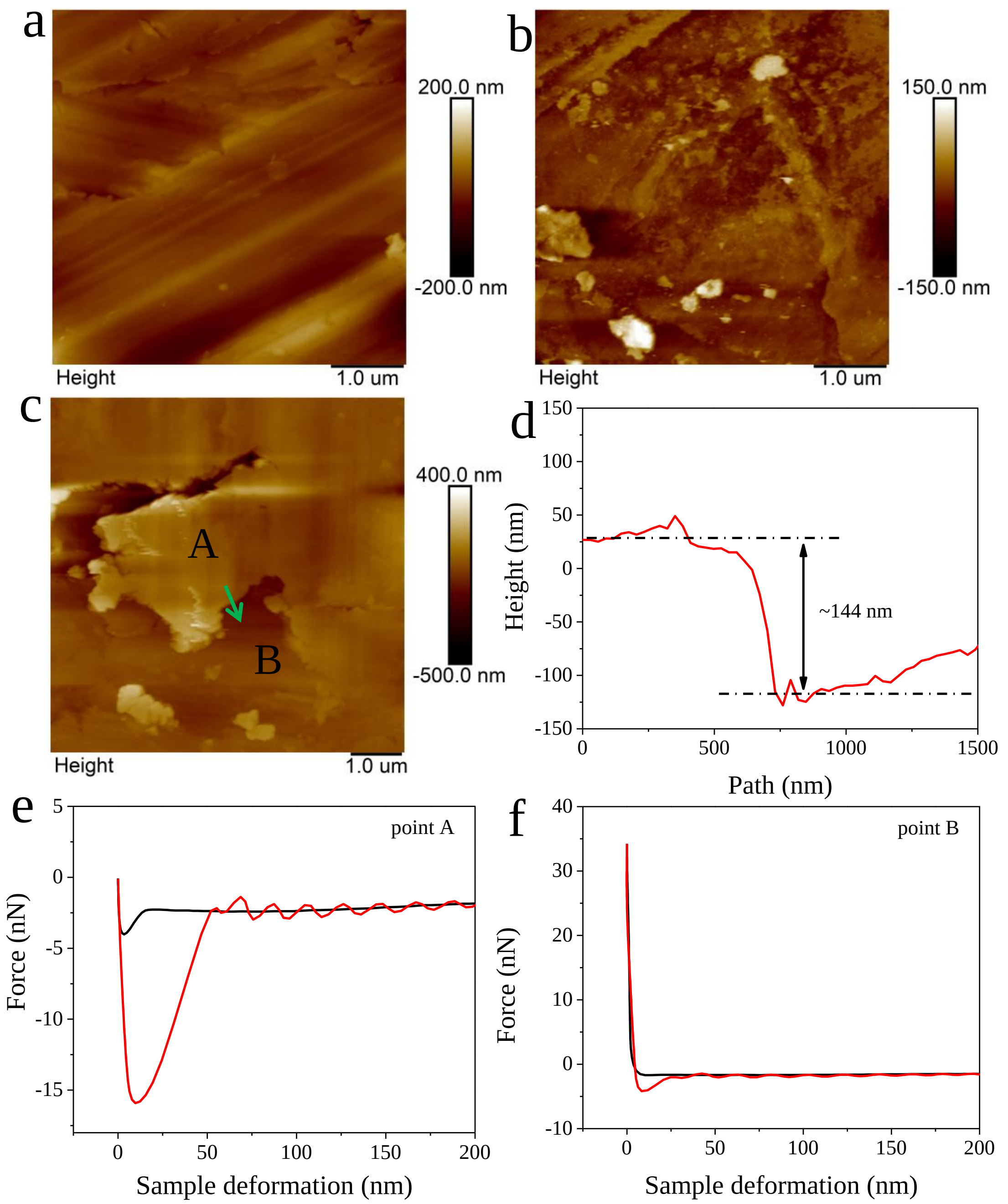

Figure S7. Morphologies of Zn, related to Figure 2.

AFM characterization. (a) Pristine Zn plate, Zn plates after galvanostatic cycling for 120 h in the absence of O_2 (b) and in presence of O_2 (c). (d) The corresponding section height curve of the green line in (c). Indentation curves of Zn plate at point A (e) and B (f).

For the AFM measurement, the pristine Zn surface showed a metal stripe structure

before cycling (Figure S7a). As shown in Figure S7b, there was no serious corrosion on the surface of Zn after cycling 120 h in the absence of O_2 (there was a bits of Zn dendrite), but the flatness of the surface of Zn slightly changed. This was due to the slight corrosion on the surface of Zn. However, as shown in Figure S7c, the surface of Zn was severely corroded after cycling for 120 h in the presence of O_2. The corresponding section height curve showed that the corrosion depth was about 144 nm (Figure S7d). As shown in Figure S7c, point A was the surface of Zn that was not severely corroded and point B was the surface of severely corroded Zn. In addition, the mechanical properties of point A and point B was measured.(Shen et al., 2019) The penetration depth of the probe at point A (Figure S7e) was significantly higher than point B (Figure S7f). The corresponding peak force and the reduced modulus of Zn plate in the presence of O_2 were shown in Table S2. The modulus at point B of the corrosion region was significantly higher than that at point A, which was about one order of magnitude higher. It further reflected that O_2 accelerated the corrosion on the Zn surface, thereby generating some by-products such as zinc oxide, zinc hydroxide and zincate.

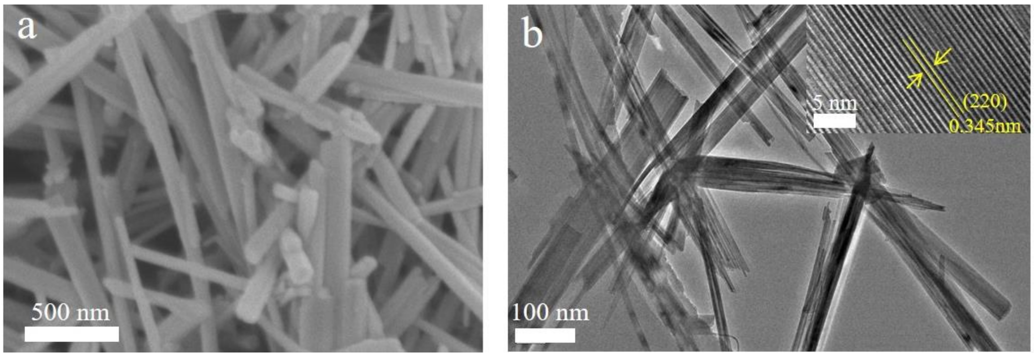

Figure S8. Characterization of α-MnO_2, related to Figure 3.

Structural characterization of α-MnO_2. (a) SEM image. (b) TEM characterization, and the inset is HRTEM image.

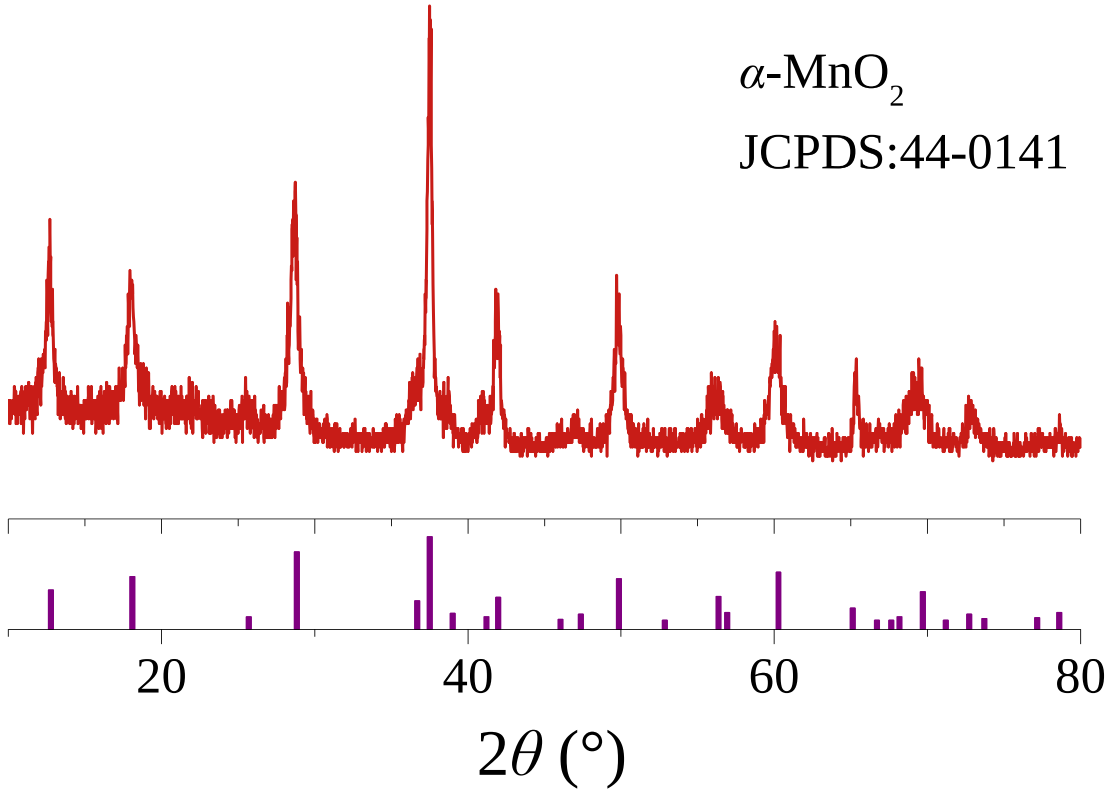

Figure S9. Characterization of α-MnO_2, related to Figure 3.

XRD data of α-MnO_2.

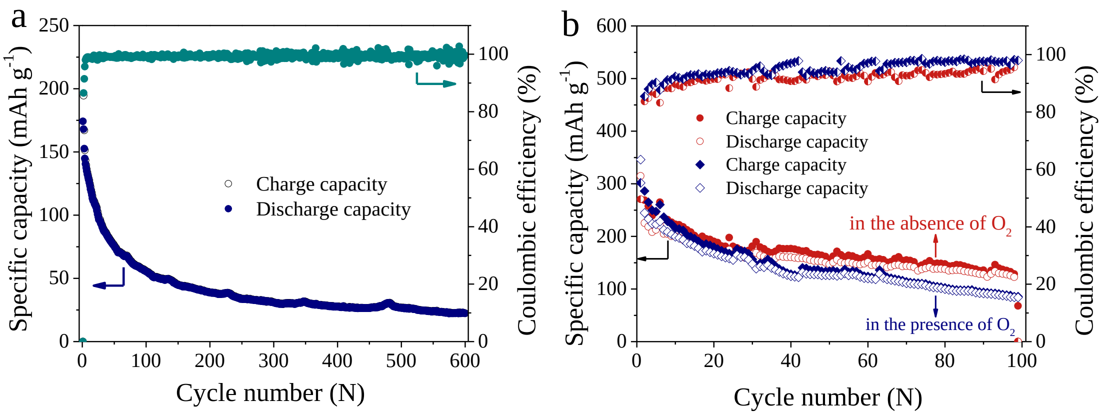

Figure S10. Electrochemical study of Zn/MnO_2 batteries, related to Figure 3.

(a) The cycling performance of the aqueous Zn/MnO_2 battery using 2 M $ZnSO_4$ electrolyte in the open laboratory environment (the content of dissolved O_2 was 6.24 mg L^{-1}) at 1C (0.308 A g^{-1}). (b) Cycling stability test of the aqueous Zn/MnO_2 batteries using 2 M $ZnSO_4$ electrolytes in the presence of O_2 and in absence of O_2 at 0.1 A g^{-1} with voltage window of 1~1.9 V.

When the voltage window of Zn-MnO_2 battery was limited within 1~1.8 V, there will be less oxygen generated via OER at the cathode side. Even though we have verified the α-MnO_2 electrode exhibited a smaller overpotential (58 mV) at 5 mA cm^{-2} in the presence of O_2 than that in the absence of O_2, but the LSV characterization indicated that, the oxygen evolution reaction (OER) catalytic activity of the α-MnO_2 was unsatisfactory when compared to some typically reported OER catalysts. As for the long-term difference whether eliminate oxygen or not, we have extended the voltage window of the Zn-MnO_2 battery of 1~1.9 V, thus, the cycling stability test of the aqueous Zn/MnO_2 batteries using 2 M $ZnSO_4$ electrolytes in the presence of O_2 and in absence of O_2 at 0.1 A g^{-1} can be compared as shown in Figure S10. The corresponding Zn-MnO_2 battery exhibited a faster capacity attenuation in the O_2-rich environment. The results proved that the existence of dissolved O_2 indeed resulted in the corrosion of Zn anode, and thus aggravated the capacity decay of the Zn-MnO_2 battery.

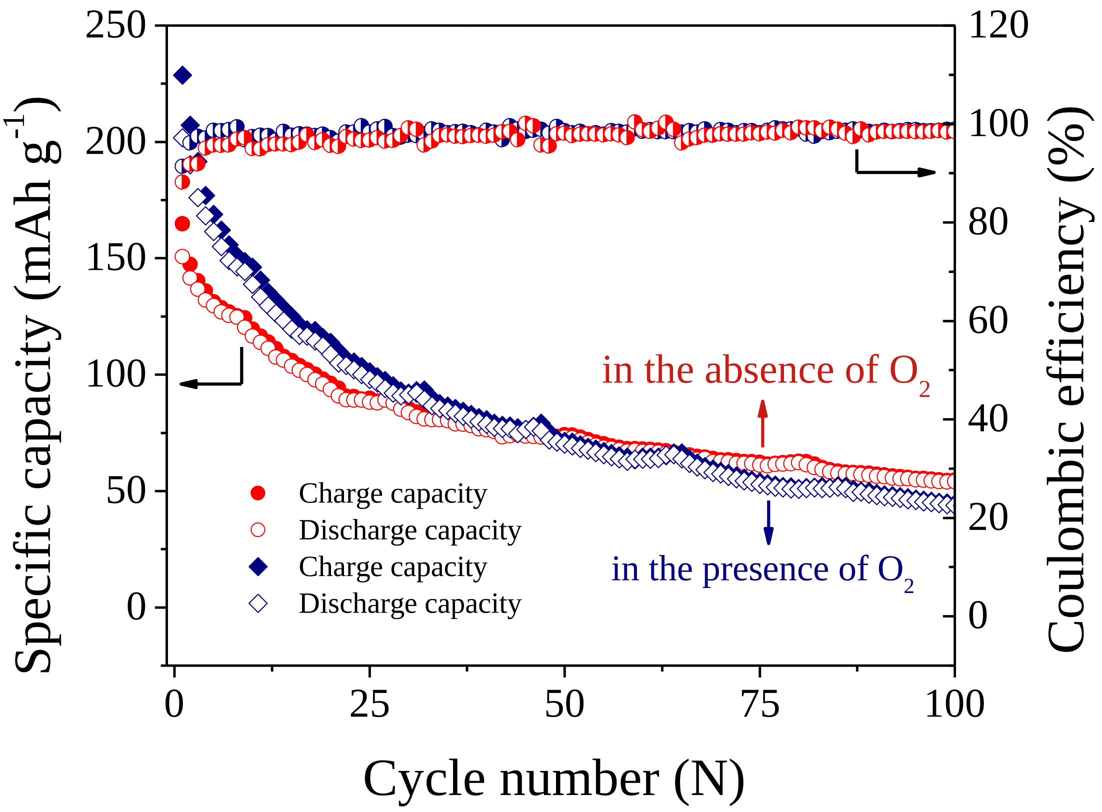

Figure S11. Electrochemical study of Zn/MnO$_2$ battery, related to Figure 3.

Cycling stability test of the aqueous Zn/MnO$_2$ batteries using 2 M ZnSO$_4$ electrolytes in the presence of O$_2$ and in absence of O$_2$ at 0.1 A g^{-1} without adding conductive graphite of cathode material.

We have compared the cycling stability of aqueous Zn/MnO$_2$ batteries using 2 M ZnSO$_4$ electrolytes in the presence of O$_2$ and in absence of O$_2$ at 0.1 A g^{-1} without adding conductive graphite of cathode material (Figure S11). A high reversible capacity was delivered in the first cycle in the presence of oxygen. Thus, the extra charge capacity was not due to the oxidation of conductive graphite.

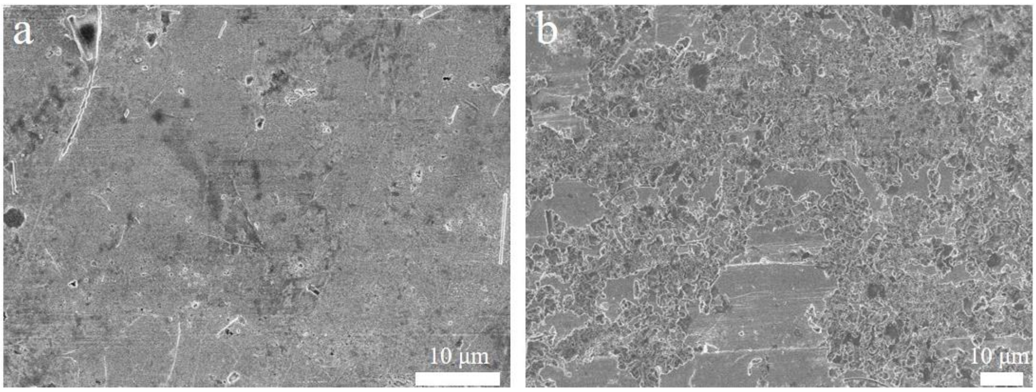

Figure S12. Characterization of Zn, related to Figure 3.

SEM images of Zn anodes for the aqueous Zn/MnO_2 batteries after cycling test (corresponding to Figure 3a) in the absence of O_2 (a) and in presence of O_2 (b).

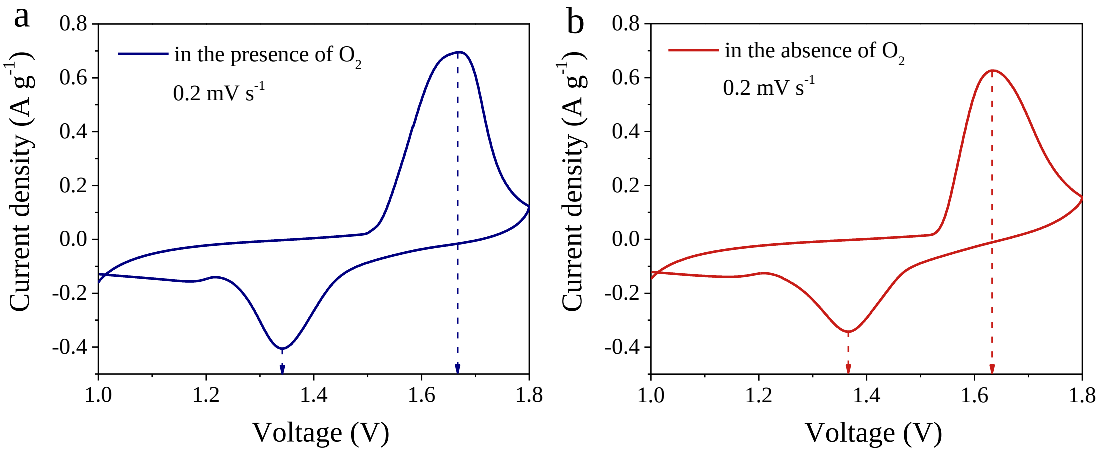

Figure S13. Electrochemical study of Zn/MnO_2 batteries, related to Figure 4.

CV curves of aqueous Zn/MnO_2 batteries at 0.2 mV s^{-1} in the presence (a) and in absence of O_2 (b).

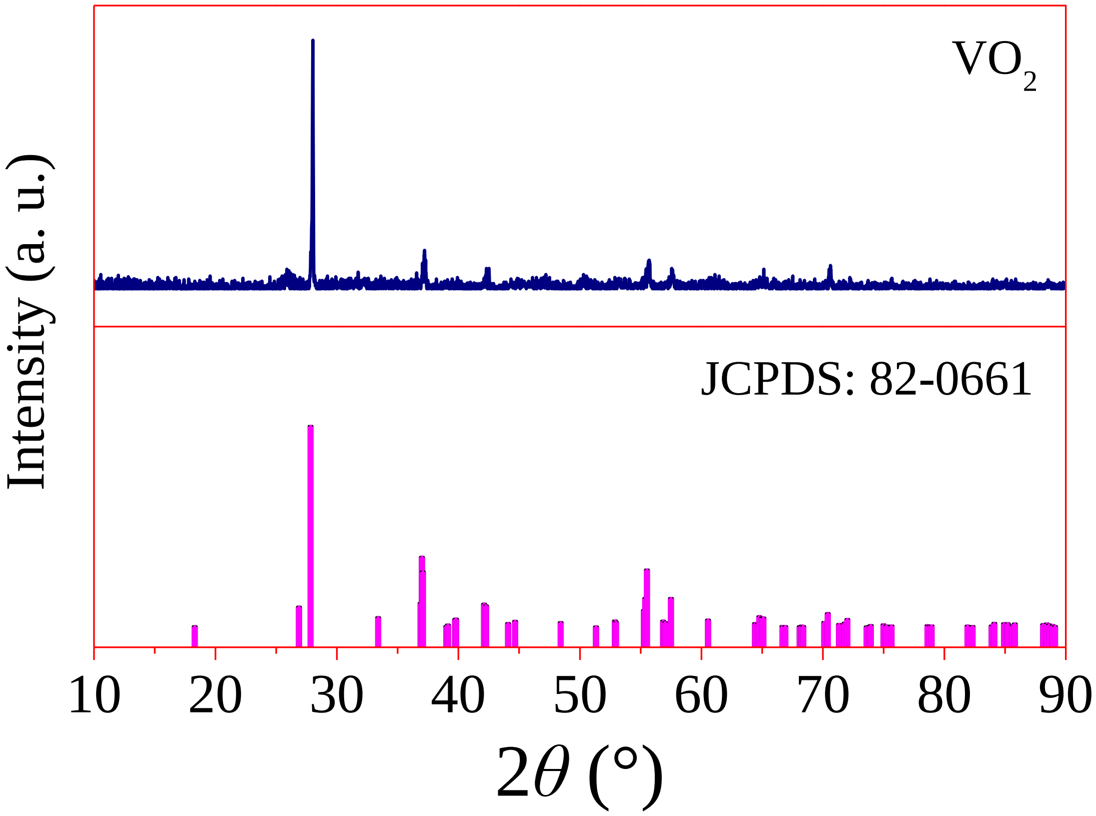

Figure S14. Characterization of VO_2, related to Figure 5.

XRD data of as-prepared VO_2.

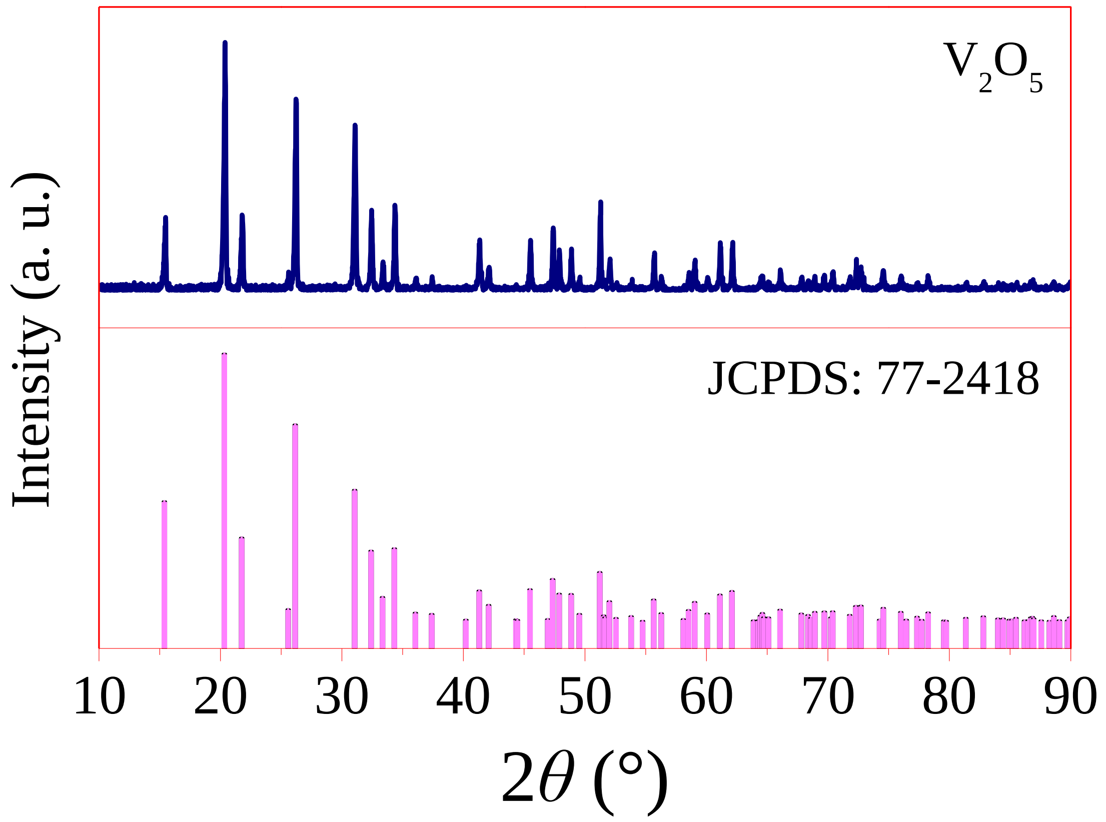

Figure S15. Characterization of V_2O_5, related to Figure 5.

XRD data of as-prepared V_2O_5.

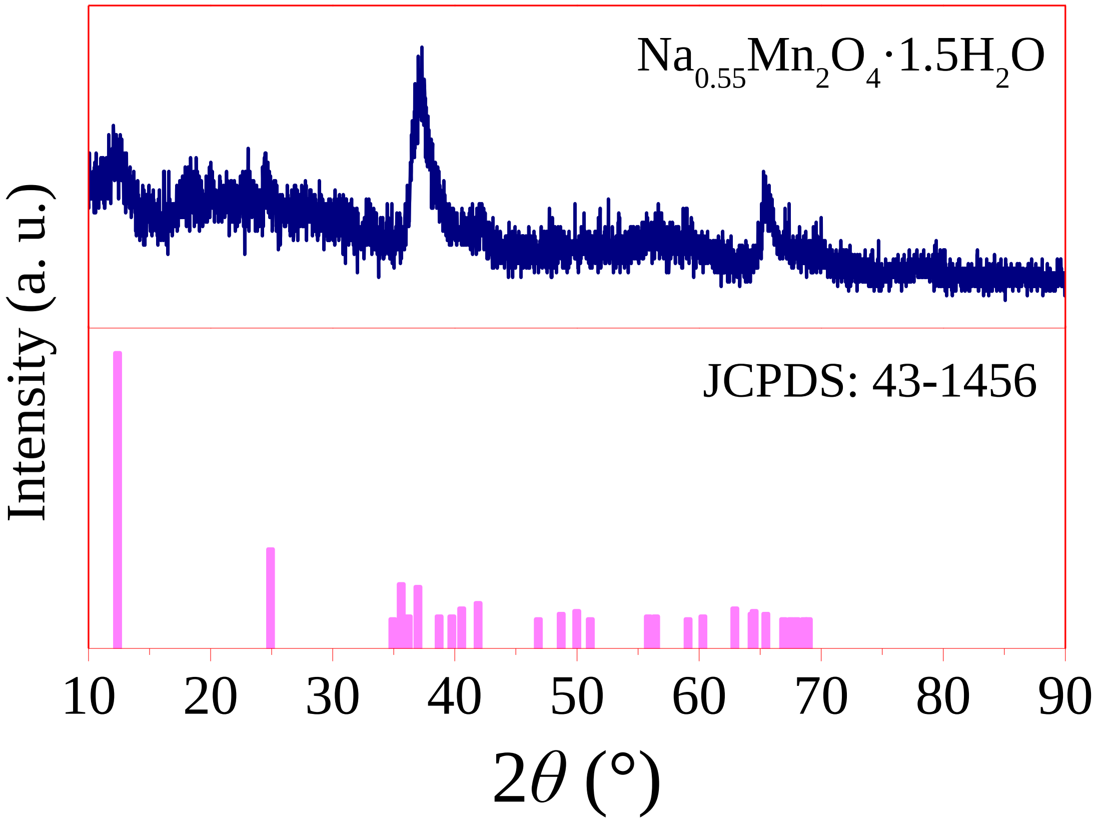

Figure S16. Characterization of $Na_{0.55}Mn_2O_4 \cdot 1.5H_2O$, related to Figure 5.

XRD data of as-prepared $Na_{0.55}Mn_2O_4 \cdot 1.5H_2O$.

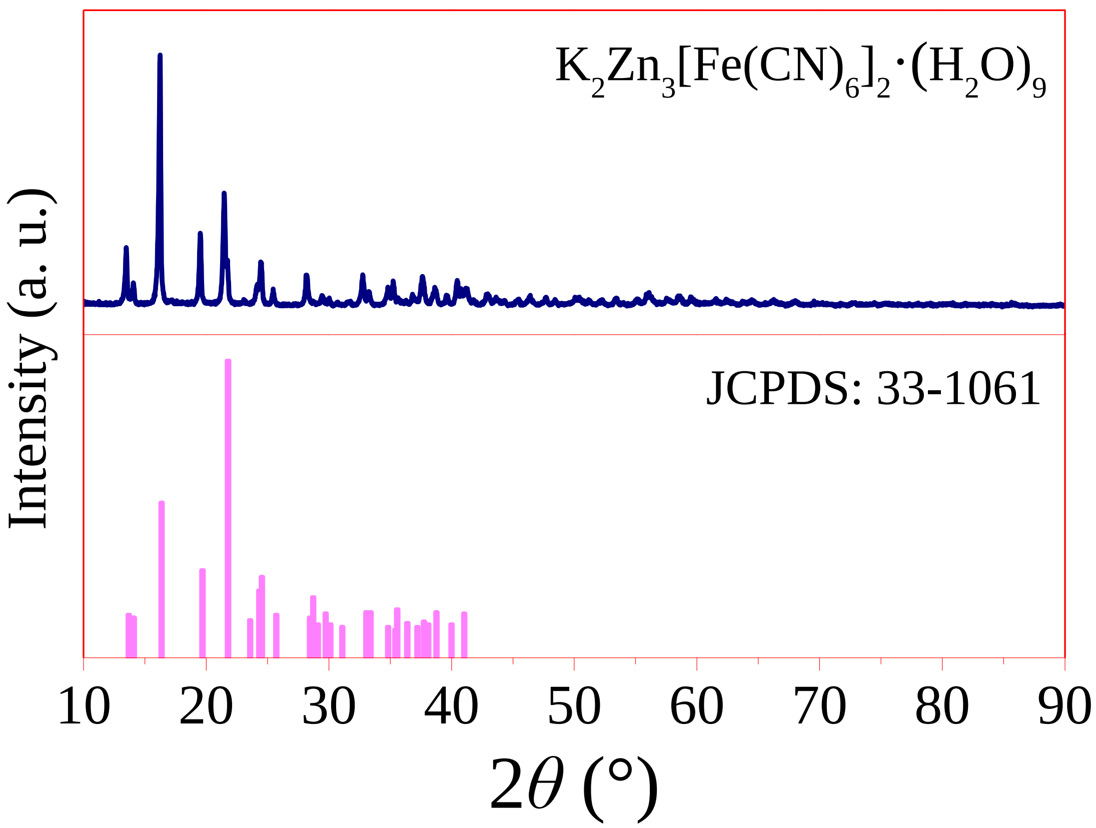

Figure S17. Characterization of $K_2Zn_3[Fe(CN)_6]_2 \cdot (H_2O)_9$, related to Figure 5.

XRD data of as-prepared $K_2Zn_3[Fe(CN)_6]_2 \cdot (H_2O)_9$.

Table S1. Electrochemical performance of Zn-based energy storage systems in $ZnSO_4$ aqueous electrolytes (containing dissolved oxygen), related to **Figure 3 and Figure 5**.

**Cathode material**	**Average discharge voltage (vs. Zn/Zn^{2+})**	**Specific capacity**	**Rate performance**	**Cycle performance**	**Ref.**
α-MnO_2	1.44 V at 61.6 mA g^{-1}	255 mAh g^{-1} at 61.6 mA g^{-1}	31.4% retained at 308 mA g^{-1}	50% retained after 15 cycles at 102.7 mA g^{-1}	(Pan et al., 2016)
α-MnO_2	1.44 V at 61.6 mA g^{-1}	255 mAh g^{-1} at 61.6 mA g^{-1}	31.4% retained at 308 mA g^{-1}	17.6% retained after 400 cycles at 61.6 mA g^{-1}	(Pan et al., 2016)
α-MnO_2	1.26 V at 83 mA g^{-1}	353 mAh g^{-1} at 16 mA g^{-1}	12.2% retained at 1333 mA g^{-1}	63% retained after 50 cycles at 83 mA g^{-1}	(Alfaruqi et al., 2015)
α-MnO_2	1.26 V at 10.5 mA g^{-1}	205 mAh g^{-1} at 105. mA g^{-1}	57.7% retained at 210 mA g^{-1}	66% retained after 30 cycles at 10.5 mA g^{-1}	(Lee et al., 2014)
α-MnO_2	1.3 V at 10.5 mA g^{-1}	195 mAh g^{-1} at 10.5 mA g^{-1}	85.6% retained at 42 mA g^{-1}	70% retained after 30 cycles at 42 mA g^{-1}	(Boeun et al., 2015)
α-MnO_2	1.3 V at 16	323 mAh	14.6% retained	46% retained after	(Alfaruqi

	mA g^{-1}	g^{-1} at 16 mA g^{-1}	at 1666 mA g^{-1}	75 cycles at 83 mA g^{-1}	et al., 2016)
γ-MnO_2	1.32 V at 0.05 mA cm^{-2}	285 mAh g^{-1} at 0.05 mA cm^{-2}	57.8% retained at 0.5 mA cm^{-2}	63% retained after 40 cycles at 0.5 mA cm^{-2}	(Alfaruqi et al., 2015)
Layered MnO_2	1.3 V at 50 mA g^{-1}	289 mAh g^{-1} at 50 mA g^{-1}	21.1% retained at 1000 mA g^{-1}	35% retained after 50 cycles at 100 mA g^{-1}	(Alfaruqi et al., 2018)
δ- MnO_2	1.28 V at 83 mA g^{-1}	250 mAh g^{-1} at 83 mA g^{-1}	24.6% retained at 1333 mA g^{-1}	46% retained after 100 cycles at 83 mA g^{-1}	(Alfaruqi et al., 2015)
δ- MnO_2	0.75 V at 12.3 mA g^{-1}	120 mAh g^{-1} at 12.3 mA g^{-1}	27.3% retained at 308 mA g^{-1}	48% retained after 125 cycles at 12.3 mA g^{-1}	(Han et al., 2017)
LiV_3O_8	0.81 V at 16 mA g^{-1}	256 mAh g^{-1} at 16 mA g^{-1}	11.3% retained at 1666 mA g^{-1}	75% retained after 65 cycles at 133 mA g^{-1}	(Alfaruqi et al., 2017)
$Zn_3V_2O_7(OH)_2·2H_2O$	0.72 V at 50 mA g^{-1}	213 mAh g^{-1} at 50 mA g^{-1}	25.4% retained at 3000 mA g^{-1}	68% retained after 300 cycles at 200 mA g^{-1}	(Xia et al., 2017)
$V_{1-x}Al_xO_{1.52}(OH)_{0.77}$	0.65 V at 15 mA g^{-1}	156 mAh g^{-1} at 15	41.0% retained at 1000 mA g^{-1}	68% retained after 50 cycles at 15 mA	(Jo et al., 2017)

		mA g^{-1}		g^{-1}	
$V_3O_7·H_2O$	0.72 V at 375 mA g^{-1}	375 mAh g^{-1} at 1 C	_	80 % retained after 200 cycles	(Kundu et al., 2018)
α-MnO_2	1.44 V at 100 mA g^{-1}	202 mAh g^{-1} at 0.1 A g^{-1}	_	38% retained after 100 cycles at 0.1 A g^{-1}	**This work**
$Na_{0.55}Mn_2O_4·1.5H_2O$	1.39 V at 100 mA g^{-1}	107 mAh g^{-1} at 0.1 A g^{-1}	_	32% retained after 100 cycles at 0.1 A g^{-1}	
VO_2	0.68 V at 100 mA g^{-1}	310 mAh g^{-1} at 0.1 A g^{-1}	_	36% retained after 100 cycles at 0.1 A g^{-1}	
V_2O_5	0.82 V at 100 mA g^{-1}	191 mAh g^{-1} at 0.1 A g^{-1}	_	37% retained after 100 cycles at 0.1 A g^{-1}	
$K_2Zn_3[Fe(CN)_6]_2·(H_2O)_9$	1.03 V at 100 mA g^{-1}	65 mAh g^{-1} at 0.1 A g^{-1}	_	43% retained after 100 cycles at 0.1 A g^{-1}	

Table S2. The peak force and the reduced modulus of Zn plate in the presence of O_2, related to **Figure 2**.

Interfacial layer	Peak force (nN)	Reduce Modulus
Zn oxides/hydroxides (Point B)	2.8	~10 GPa
Zn (Point A)	13.7	~600 MPa

Supplementary References

Alfaruqi, M. H., Gim, J,. Kim, S., Song, J., Jo, J., Kim, S., Mathew, V., and Kim, J. (2015) Enhanced reversible divalent zinc storage in a structurally stable α-MnO_2 nanorod electrode. J. Power Sources *288,* 320-327.

Alfaruqi, M. H., Gim, J., Kim, S., Song, J., Pham, D. T., Jo, J., Xiu, Z., Mathew, V., and Kim, J. (2015) A layered δ-MnO_2 nanoflake cathode with high zinc-storage capacities for eco-friendly battery applications. Electrochem. Commun. *60,* 121-125.

Alfaruqi, M. H., Islam, S., Gim, J., Song, J., Kim, S., Pham, D. T., Jo, J., Xiu, Z., Mathew, V., and Kim, J. (2016) A high surface area tunnel-type α-MnO_2 nanorod cathode by a simple solvent-free synthesis for rechargeable aqueous zinc-ion batteries. Chem. Phys. Lett. *650,* 64-68.

Alfaruqi, M. H., Islam, S., Putro, D. Y., Mathew, V., Kim, S., Jo, J., Kim, S., Sun, Y. K., Kim, K., and Kim, J. (2018) Structural transformation and electrochemical study of layered MnO_2 in rechargeable aqueous zinc-ion battery. Electrochim. Acta *276,* S0013468618308995.

Alfaruqi, M. H., Mathew, V., Gim, J., Kim, S., Song, J., Baboo, J. P., Choi, S. H., and Kim, J. (2015) Electrochemically Induced Structural Transformation in a γ-MnO_2 Cathode of a High Capacity Zinc-Ion Battery System. Chem. Mater. *27,* 3609-3620.

Alfaruqi, M. H., Mathew, V., Song, J., Kim, S., Islam, S., Pham, D. T., Jo, J., Kim, S., Baboo, J. P., and Xiu, Z. (2017) Electrochemical Zinc Intercalation in Lithium Vanadium Oxide: A High-Capacity Zinc-Ion Battery Cathode. Chem. Mater. *29,* 1684-1694.

Boeun, L., Hae, Ri L., Haesik, K., Kyung, Yoon C., Byung, Won. C., and Hyoung, O. S. (2015) Elucidating the intercalation mechanism of zinc ions into α-MnO_2 for

rechargeable zinc batteries. Chem. Commun. *51,* 9265-9268.

Ding, J. W., Du, Z. G., Gu, L. Q., Li, B., Wang, L. Z., Wang, S. W., Gong, Y. J., and Yang, S. B. (2018) Ultrafast Zn^{2+} Intercalation and Deintercalation in Vanadium Dioxide. Adv. Mater. *30,* 6.

Han, S. D., Kim, S., Li, D., Petkov, V., Yoo, H. D., Phillips, P. J., Wang, H., Kim, J. J., More, K. L., and Key, B. (2017) Mechanism of Zn Insertion into Nanostructured δ-MnO2: A Nonaqueous Rechargeable Zn Metal Battery. Chem. Mater. *29,* 4874-4884.

Hu, Z. M., Xiao, X., Jin, H. Y., Li, T. Q., Chen, M., Liang, Z., Guo, Z. F., Li, J., Wan, J., Huang, L., Zhang, Y. R., Feng, G., and Zhou, J. (2017) Rapid mass production of two-dimensional metal oxides and hydroxides via the molten salts method. Nat. Commun. *8,* 9.

Jo, J. H., Sun, Y-K., and Myung, S-T. (2017) Hollandite-type Al-doped $VO_{1.52}(OH)_{0.77}$ as a zinc ion insertion host material. J. Mater. Chem. A *5,* 8367-8375.

D. Kundu, S. H. Vajargah, L. Wan, B. Adams, D. Prendergast and L. F. Nazar, (2018) Aqueous vs. nonaqueous Zn-ion batteries: consequences of the desolvation penalty at the interface. *Energy Environ. Sci. 11,* 881-892.

Lee, B., Yoon, C. S., Lee, H. R., Chung, K. Y., Cho, B. W., and Oh, S. H. (2014) Electrochemically-induced reversible transition from the tunneled to layered polymorphs of manganese dioxide. Sci. Rep. *4,* 6066.

Pan, H., Shao, Y., Yan, P., Cheng, Y., Han, K. S., Nie, Z., Wang, C., Yang, J., Li, X., and Bhattacharya, P. (2016) Reversible aqueous zinc/manganese oxide energy storage from conversion reactions. Nat. Energy *1,* 16039.

Shen, X., Li, Y., Qian, T., Liu, J., Zhou, J., Yan, C., and Goodenough, J. B. (2019) Lithium anode stable in air for low-cost fabrication of a dendrite-free lithium battery. Nat. Commun. *10,* 900.

Xia, C., Guo, J., Lei, Y., Liang, H., Zhao, C., and Alshareef, H. N. (2017) Rechargeable Aqueous Zinc-Ion Battery Based on Porous Framework Zinc Pyrovanadate Intercalation Cathode. Adv. Mater. *30,* 1705580.

Zhang, L., Chen, L., Zhou, X., and Liu, Z. (2015) Towards High-Voltage Aqueous Metal-Ion Batteries Beyond 1.5 V: The Zinc/Zinc Hexacyanoferrate System. Adv.

Energy Mater. *5,* 1400930.

Zhang, N., Dong, Y., Jia, M., Bian, X., Wang, Y. Y., Qiu, M. D., Xu, J. Z., Liu, Y. C., Jiao, L. F., and Cheng, F. Y. (2018) Rechargeable Aqueous Zn-V_2O_5 Battery with High Energy Density and Long Cycle Life. ACS Energy Lett. *3,* 1366-1372.
